# Novel Silver(I) and Gold(I) N‐Heterocyclic Carbene Complexes Induce ROS‐Dependent Autophagic Cell Death in Human Hepatoma Cell Line HepG2

**DOI:** 10.1111/cbdd.70283

**Published:** 2026-03-27

**Authors:** Rocchina Miglionico, Francesco Viceconte, Maria Francesca Armentano, Annaluisa Mariconda, Ilaria Nigro, Pasquale Longo, Faustino Bisaccia

**Affiliations:** ^1^ Department of Health Sciences University of Basilicata Potenza Italy; ^2^ Department of Basic and Applied Sciences University of Basilicata Potenza Italy; ^3^ Department of Chemistry and Biology University of Salerno Fisciano Italy

**Keywords:** AKT/mTOR pathway, apoptosis resistance, autophagy, hepatocellular carcinoma, reactive oxygen species, silver(I) and gold(I) NHC complexes

## Abstract

Hepatocellular carcinoma is one of the most aggressive malignancies worldwide, with limited treatment options and high resistance to conventional therapies. Developing novel therapeutic strategies that target alternative cell death mechanisms is crucial for overcoming treatment resistance. This study evaluated the cytotoxicity of eight sulfonated silver(I) and gold(I) N‐heterocyclic carbene (NHC) complexes—four newly synthesized—against human liver cancer cells and investigated the mechanisms of the compounds that exhibited higher selectivity for cancer cells compared to non‐malignant liver cells. Morphological analysis revealed distinct features of autophagy rather than apoptosis, as confirmed by the absence of chromatin condensation, caspase‐3 activation, and PARP‐1 cleavage. Instead, both complexes strongly upregulated Beclin‐1 and LC3‐II expression—key autophagy markers—while inhibiting the AKT/mTOR signaling pathway. The observed cytotoxic effects were associated with a significant increase in reactive oxygen species (ROS) production. Pre‐treatment with the antioxidant N‐acetyl‐L‐cysteine completely abolished both cytotoxicity and autophagy induction. These findings demonstrate that silver(I) and gold(I) NHC complexes induce ROS‐dependent autophagic cell death in this kind of cancer cells. The ability of these compounds to trigger non‐apoptotic cell death mechanisms highlights their potential as promising candidates for overcoming apoptosis resistance in HCC therapy, warranting further in vivo investigations.

## Graphical abstract

In this research work, the cytotoxicity of eight sulfonated silver(I) and gold(I) N‐heterocyclic carbene (NHC) complexes—four newly synthesized—was evaluated against human liver cancer cells. Two compounds showed selectivity for cancer cells, inducing ROS‐dependent autophagic cell death via AKT/mTOR pathway inhibition without triggering apoptosis.

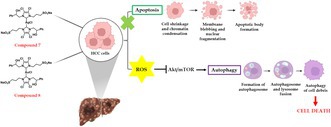

## 1 Introduction

Liver cancer was ranked as the sixth most common malignancy and the third leading cause of cancer‐related deaths worldwide in 2020 (Sung et al. 2021). Liver cancer is one of the most common tumors, and hepatocellular carcinoma (HCC) represents the most widespread form, accounting for over 80% of cases. HCC primarily develops in patients with cirrhosis, which can result from chronic infections such as hepatitis B and hepatitis C, metabolic syndrome, or alcohol abuse (Llovet et al. 2021). It is an aggressive tumor with an uncertain prognosis, often asymptomatic in its early stages, leading to diagnosis at an advanced phase. Treatment options require a multidisciplinary approach and depend on the tumor stage, the degree of hepatic impairment, and the patient's overall condition. Early‐stage HCC can be treated successfully with surgical resection, ablation, or liver transplantation, whereas advanced‐stage HCC is managed with systemic therapy (Salgia and Mendiratta 2021; Siddique et al. 2017). In the past, systemic chemotherapy, including cisplatin, doxorubicin, etoposide, 5‐fluorouracil (5‐FU), and their combinations, demonstrated limited efficacy (Cao et al. 2012). From 2007 to 2017, sorafenib, a tyrosine kinase inhibitor (TKI), was the only first‐line treatment approved by the U.S. Food and Drug Administration (FDA). Subsequently, new therapeutic agents have been introduced, including other TKIs, monoclonal antibodies, and immune checkpoint inhibitors (ICIs) (Fan et al. 2022). Given that HCC development is a complex process mediated by multiple pathways, several combination chemotherapy regimens have been developed, demonstrating greater efficacy than single‐agent treatments. Nevertheless, systemic therapy remains unsatisfactory, as it is often limited by the development of drug resistance and high cytotoxicity, which merely slow tumor progression and prolong patient survival (Demir et al. 2021; Ghaziani and Dhanasekaran 2021). Following the introduction of cisplatin and other platinum‐based drugs into clinical practice for the treatment of solid tumors, including HCC, breast, lung, and kidney cancers (Dasari et al. 2022; Dasari and Tchounwou 2014), several metal complexes were synthesized and investigated for their potential antitumor activity (Lucaciu et al. 2022; Ndagi et al. 2017).

Among the various metal‐based compounds tested, silver (Ag) and gold (Au) complexes play a significant role. Initially, they were used as antimicrobial and anti‐arthritis drugs, respectively, but later demonstrated notable anti‐tumor activity. Silver and gold ions can be efficiently stabilized by cyclic carbenes, such as N‐Heterocyclic carbenes (NHCs). These versatile neutral ligands are widely utilized in the design of efficient catalysts (Nesterov et al. 2018), as their stereoelectronic properties can be easily fine‐tuned by selecting appropriate ring substituents. Several studies have shown that silver and gold NHC complexes exhibit a variety of biological properties, notably anti‐inflammatory, antioxidant, antibacterial, anticancer, and antiviral activities (Achar et al. 2019; Guarra et al. 2021; Iqbal et al. 2015; Mora et al. 2019). Moreover, their structures and hydrophilic/lipophilic properties can be readily modified, making them a promising approach for the development of metal‐containing drugs.

**FIGURE 1:**
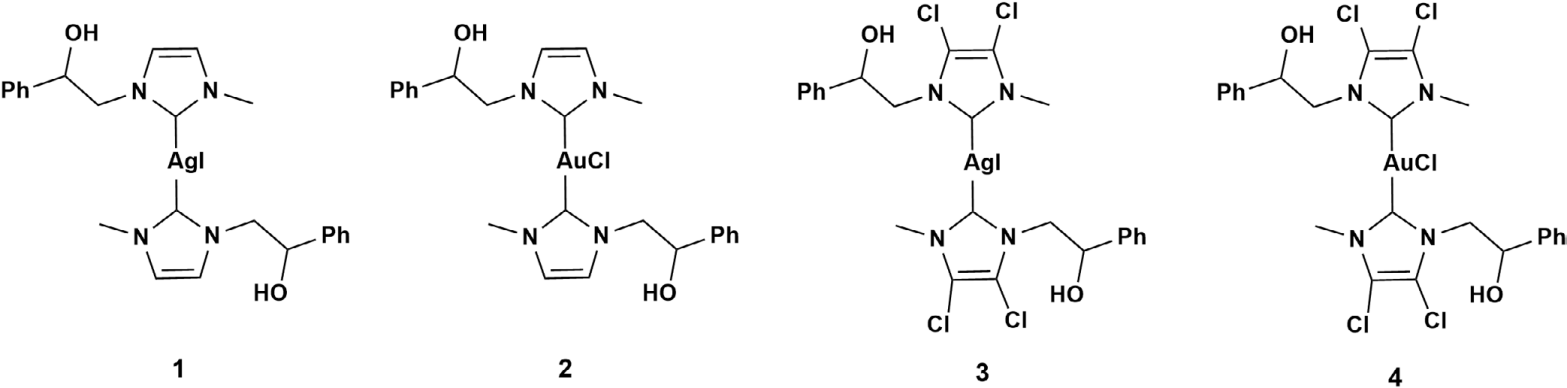
Structures of previously synthesized Ag(I) and Au(I) NHC complexes.

Some of us have synthesized several silver(I) and gold(I)‐based complexes stabilized by NHC ligands and studied their antibacterial and antitumor properties. Figure 1 shows the structures of the most promising complexes (**1**–**4**).

Complex **1** demonstrated antimicrobial activity, inhibiting the growth of *E. coli* and *B. subtilis* at a concentration of 5 mg/mL (Napoli et al. 2013). Compound **2** (Saturnino et al. 2016) showed potent activity against breast cancer cells, with IC50 values of 1 μM (95% CI = 0.8–1.2) for MCF‐7 and 2.6 μM (95% CI = 2.2–3.2) for ZR‐75‐1 cells. Furthermore, compounds **3** and **4** (Iacopetta et al. 2020) were particularly effective against MDA‐MB‐231 cancer cells, exhibiting IC50 values of 3.22 ± 1.2 μM and 2.1 ± 0.7 μM, respectively. The structure of these complexes is characterized by the asymmetric substitution of nitrogen atoms and the presence of hydrogen or chlorine atoms on the NHC backbone.

**FIGURE 2:**
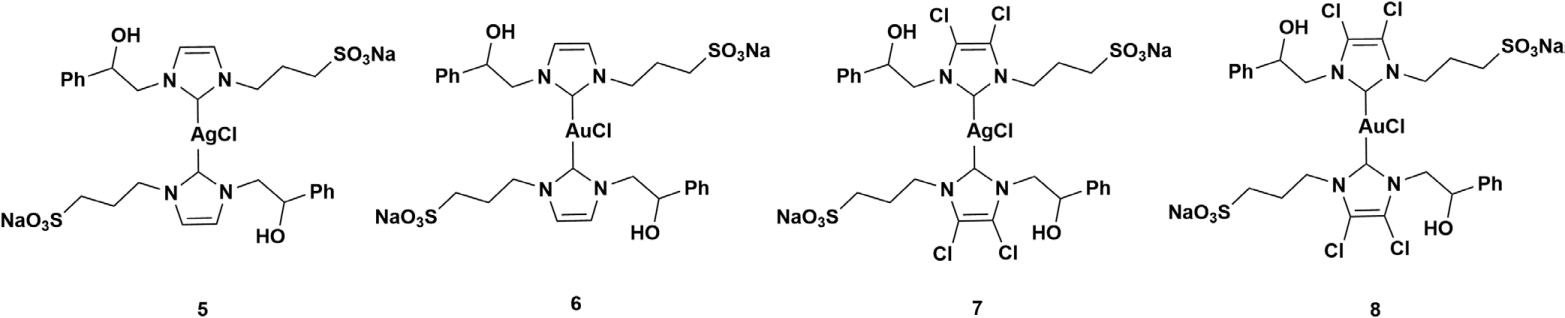
NHC silver(I) and gold(I) complexes functionalized with a sulfonate group.

Building on this foundation, we designed new NHC silver and gold compounds by modifying the structure of the complexes shown in Figure 1. More specifically, we replaced the methyl group with a hydrophilic ionic substituent, such as a sulfonate group (Figure 2).

The sulfonate group's introduction increases these complexes' water solubility, which is advantageous for applications in physiological settings (Gandin et al. 2013; Gürbüz et al. 2020; Johnson et al. 2017).

We specifically investigated the molecular mechanism of action of compounds **7** and **8**, which exhibited lower cytotoxicity in the healthy cell line while inducing comparable cytotoxic effects in the cancer cell lines. Through various biological assays, we demonstrated that both complexes exert their cytotoxic activity by increasing ROS production, leading to the subsequent activation of autophagy.

## 2 Materials and Methods

### 2.1 Chemistry

Chemicals, starting materials, and deuterated dimethyl sulfoxide‐*d*_6_ (99.80%) were purchased from commercial sources (Merck and TCI Chemicals) and used without further purification. NMR spectra were recorded on two types of Bruker spectrometers at different resonance frequencies: Bruker AM 300 (300 MHz for ^1^H, 75 MHz for ^13^C) and Bruker AVANCE 400 (400 MHz for ^1^H, 100 MHz for ^13^C). All chemical shifts (δ) are provided in parts per million (ppm) and were recorded relative to residual solvent peaks. Multiplicities: (s)inglet, (d)oublet, (t)riplet, unresolved (m)ultiplet, (o)verlapped, and (br)oad. The ^1^H‐ and ^13^C‐NMR spectra are reported in the Supporting Information (Figures S1–S16). Elemental analysis was conducted with a PERKIN‐Elmer 240‐C analyzer. The Bruker solariX was used to acquire MALDI‐ToF mass spectra.

### 2.2 Synthesis of 1‐(2‐Hydroxy‐2‐Phenylethyl)‐1H‐Imidazole (A) and 1‐(2‐Hydroxy‐2‐Phenylethyl)‐1H‐4,5‐Dichloroimidazole (B)

Compounds 1‐(2‐hydroxy‐2‐phenylethyl)‐1H‐imidazole (A) and 1‐(2‐hydroxy‐2‐phenylethyl)‐1H‐4,5‐dichloroimidazole (B) were prepared according to literature methods with a yield of 57% and 45%, respectively (Napoli et al. 2013; Mariconda et al. 2020; Ceramella et al. 2023; D'Amato et al. 2024).

- Characterization ^1^H‐NMR (400 MHz, DMSO‐*d*_*6*_, ẟ ppm): 7.45 (s, 1H, NC*H*N), 7.28 (m, 5H, aromatic protons), 7.05 (s, 1H, CH_2_NC*H*CHN), 6.78 (s, CH_2_NCHC*H*N, 1H), 5.74 (br, 1H, O*H*), 4.78 (m, 1H, C*H*OH), 4.18–3.98 (m, 2H, NC*H*_*2*_CHOH). ^13^C‐NMR (75 MHz, DMSO‐*d*_*6*_, ẟ ppm): 142.6 (ipso aromatic carbon), 137.7 (N*C*HN), 128.0–127.7‐127.3‐126.0 (aromatic carbons), 120.0 (N*C*H*C*HN), 72.0 (*C*HOH), 53.5 (N*C*H_2_).
- Characterization ^1^H‐NMR (300 MHz, DMSO‐*d*_*6*_, ẟ ppm): 7.69 (s, 1H, NC*H*N) 7.34–7.31 (m, 5H, aromatic protons), 5.83 (d, 1H, O*H*), 4.86 (m, 1H, C*H*OH), 4.10 (m, 2H, NC*H*_*2*_CHOH). ^13^C‐NMR (75 MHz, DMSO‐*d*_*6*_, ẟ ppm): 141.72 (ipso aromatic carbon), 136.7 (N*C*HN), 128.2, 127.6, 125.9, (aromatic carbons), 123.7 and 112.4 (N*C*Cl*C*ClN), 70.7 (*C*HOH), 52.7 (N*C*H_2_CHOH).

### 2.3 Synthesis of Sulfonated Imidazolinium Pro‐Ligands 3‐(3‐(2‐Hydroxy‐2‐Phenylethyl)‐1H‐Imidazol‐3‐Ium‐1‐Yl)propane‐1‐Sulfonate (S_A_ ) and 3‐(4,5‐Dichloro‐3‐(2‐Hydroxy‐2‐Phenylethyl)‐1H‐Imidazol‐3‐Ium‐1‐Yl)Propane‐1‐Sulfonate (S_B_ )

The A or B mono‐alkylate product (1.0 mmol) was dissolved in acetonitrile (11 mL), and 1,3‐propane sultone (1.1 mmol) was added. The mixture underwent reflux for 24 h (A) or 48 h (B). Then the solvent was removed. The residue was then suspended in a minimum amount of acetone and filtered. After filtration, the salt was dried under vacuum, resulting in a final white powder. Yield: (S_A_) 92% and (S_B_) 41%.

(S_A_) characterization ^1^H‐NMR (400 MHz, DMSO‐*d*_*6*_, ẟ ppm): 9.10 (s, 1H, NC*H*N), 7.77 (s, 1H, CHCH_2_NC*H*CHNCH_2_CH_2_), 7.68 (s, 1H, CHCH_2_NCHC*H*NCH_2_CH_2_), 7.39 (m, 5H, aromatic protons), 4.94 (m, 1H, C*H*OH), 4.42–4.19 (m, 4H, CHC*H*_*2*_NCH and NC*H*_*2*_CH_2_CH_2_), 2.11 (t, 2H, NCH_2_CH_2_C*H*_*2*_), 2.07 (m, 2H, NCH_2_C*H*_*2*_CH_2_). ^13^C‐NMR (100 MHz, DMSO‐*d*_*6*_, ẟ ppm): 141.6 (ipso aromatic carbon), 137.0 (N*C*HN), 128.7, 128.2, 126.3 (aromatic carbons), 123.6 and 122.4 (N*C*H*C*HN), 71.0 (*C*HOH), 56.2 (N*C*H_2_CHOH), 48.2 (N*C*H_2_CH_2_CH_2_), 47.7 (NCH_2_CH_2_ *C*H_2_), 26.7 (NCH_2_ *C*H_2_CH_2_). Elemental Analysis: calculated for C_14_H_18_N_2_O_4_S C, 54.18; H, 5.85; N, 9.03. Found C, 54.19; H, 5.85; N, 9.02. (MALDI‐ToF, CH_3_OH): m/z = 309.16020 Da for [C_14_H_17_N_2_O_4_S]^+^.

(S_B_) characterization ^1^H‐NMR (300 MHz, DMSO‐*d*_*6*_, ẟ ppm): 9.51 (s, 1H, NC*H*N), 7.39 (m, 5H, aromatic protons), 6.08 (d, 1H, O*H*), 4.94 (m, 1H, C*H*OH), 4.43–4.27 (m, 4H, NC*H*_*2*_CHOH and NC*H*_*2*_CH_2_), 2.52 (m, 2H, NCH_2_CH_2_C*H*_*2*_), 2.09 (m, 2H, NCH_2_C*H*_*2*_CH_2_). ^13^C‐NMR (100 MHz, DMSO‐*d*_*6*_, ẟ ppm): 140.3 (ipso aromatic carbon), 137.1 (N*C*HN), 128.5, 128.0, 125.9 (aromatic carbons), 119.1 and 118.2 (N*C*Cl*C*ClN), 69.8 (*C*HOH), 55.0 (N*C*H_2_CHOH), 47.6 (NCH_2_CH_2_ *C*H_2_), 47.1 (NCH_2_CH_2_ *C*H_2_), 24.8 (NCH_2_ *C*H_2_CH_2_). Elemental Analysis: calculated for C_14_H_16_Cl_2_N_2_O_4_S C, 44.34; H, 4.25; N, 7.39. Found C, 44.39; H, 4.18; N, 7.45.

(MALDI‐ToF, CH_3_OH): m/z = 378.02073 Da attributable to [C_12_H_16_Cl_2_N_2_O_4_S]^+^.

### 2.4 Synthesis of Bis‐(NHC) Sulfonated Silver(I) Complexes: Disodium Bis(1‐(2‐Hydroxy‐2‐Phenylethyl)‐3‐(3‐Sulfonatopropyl)‐2,3‐Dihydro‐1H‐Imidazol‐2‐Yl)Silver Chloride (5) and Sodium Bis(4,5‐Dichloro‐1‐(2‐Hydroxy‐2‐Phenylethyl)‐3‐(3‐Sulfonatopropyl)‐2,3‐Dihydro‐1H‐Imidazol‐2‐Yl)Silver(I) Chloride (7)

In 30 mL water, silver oxide (1.60 mmol) was added to a solution of ligand precursor *S*_A_ or S_B_ (1.60 mmol) and heated to 80°C. The mixture reaction was swirled for 24 or 48 h, respectively, while being covered with aluminum foil. Then, the mixture was cooled at room temperature and NaCl (1.60 mmol) was added, leaving under stirring for 1 h more. Subsequently, the solvent was removed under vacuum after the dark suspension was filtered through celite to remove the excess Ag_2_O. Diethyl ether was used to wash the residue, resulting in a beige, dusty solid. Yield: (5) 59% and (7) 88%.

(**5**) characterization ^1^H‐NMR (400 MHz, DMSO‐*d*_*6*_, ẟ ppm): 7.49–7.21 (m, 7H, aromatic protons + NC*H*C*H*N), 5.93 (s, 1H, O*H*), 4.94 (br, 1H, C*H*OH), 4.27–4.18 (m, 4H, NC*H*_*2*_CH and NC*H*_*2*_CH_2_CH_2_), 2.40 (t, 2H, NCH_2_CH_2_C*H*_*2*_), 2.06 (m, 2H, NCH_2_C*H*_*2*_CH_2_). ^13^C‐NMR (100 MHz, DMSO‐d_6_, ẟ ppm): 180.8 and 178.9 (dd, 107/109Ag, N*C*N), 142.1 (ipso aromatic carbon), 128.1, 127.4, 126.0 (aromatic carbons), 122.8 and 121.3 (N*C*H*C*HN), 72.2 (*C*HOH), 58.2 (N*C*H_2_CHOH), 49.8 (N*C*H_2_CH_2_CH_2_), 47.8 (NCH_2_CH_2_ *C*H_2_), 27.2 (NCH_2_ *C*H_2_CH_2_). Elemental Analysis: calculated for C_28_H_34_AgClN_4_Na_2_O_8_S_2_: C, 41.62; H, 4.24; N, 6.93. Found C, 41.62; H, 4.25; N, 6.92. (MALDI‐ToF, DHBA): m/z = 771.06754 Da attributable to [C_28_H_34_AgN_4_Na_2_O_8_S_2_]^+^; 749.08562 Da attributable to [C_28_H_34_AgN_4_NaO_8_S_2_]^+^; 727.10342 Da attributable to [C_28_H_35_AgN_4_O_8_S_2_]^+^.

(**7**) characterization ^1^H‐NMR (400 MHz, DMSO‐*d*_*6*_, ẟ ppm): 7.30 (m, 5H, aromatic protons), 6.10 (b, 1H, O*H*), 4.98 (br, 1H, C*H*OH), 4.37 (br, 4H, NC*H*_*2*_CHOH e NC*H*_*2*_CH_2_CH_2_), 2.57 (m, 2H, NCH_2_CH_2_C*H*_*2*_), 2.09 (br, 2H, NCH_2_C*H*_*2*_CH_2_). ^13^C‐NMR (100 MHz, DMSO‐*d*_*6*_, ẟ ppm): 183.6 and 181.6 (dd, 107/109Ag, N*C*N), 141.4 (ipso aromatic carbon), 128.3, 127.7, 126.0 (aromatic carbons), 117.5 and 116.0 (N*C*Cl*C*ClN), 71.5 (*C*HOH), 56.6 (N*C*H_2_CHOH), 48.6 (N*C*H_2_CH_2_CH_2_), 47.7 (NCH_2_CH_2_CH_2_), 26.6 (NCH_2_CH_2_ *C*H_2_). Elemental Analysis: calculated for C_30_H_38_AuClN_4_Na_2_O_8_S_2_ C, 38.95; H, 4.14; N, 6.06. Found C, 38.99; H, 4.20; N, 6.01. MALDI‐ToF (CH_3_OH, m/z) = 781.05139 Da for [C_28_H_30_Cl_4_N_4_O_5_AgS]^+^ and 401.01253 Da for [C_14_H_16_Cl_2_N_2_O_4_SNa]^+^.

### 2.5 Synthesis of Bis‐(NHC) Sulfonated Gold(I) Complexes: Disodium Bis(1‐(2‐Hydroxy‐2‐Phenylethyl)‐3‐(3‐Sulfonatopropyl)‐2,3‐Dihydro‐1H‐Imidazol‐2‐Yl)Gold(I) Chloride (6) and Bis(4,5‐Dichloro‐1‐(2‐Hydroxy‐2‐Phenylethyl)‐3‐(3‐Sulfonatopropyl)‐2,3‐Dihydro‐1H‐Imidazol‐2‐Yl)Gold(I) Chloride (8)

Gold precursor Au(SMe_2_)Cl (5.29 × 10^−2^ mmol) was added to a solution of silver complex 1 or 2 (5.29 × 10^−2^ mmol) in water (0.53 mL). For 24 h, the mixture was stirred at room temperature in the dark. The AgCl salt was subsequently removed by filtration through Celite, and the liquid phase was recovered. The solvent was removed under reduced pressure, and the crude product was washed with diethyl ether. The final complex was dried and recovered in the form of pale yellow powder. Yield: (6) 46% and (8) 43%.

(**6**) characterization ^1^H‐NMR (400 MHz, DMSO‐*d*_*6*_, ẟ ppm): 7.41–7.23 (m, 7H, aromatic protons + NC*H*C*H*N), 5.84 (d, 1H, O*H*), 5.04 (m, C*H*OH, 1H), 4.23–4.14 (m, 4H, NC*H*_*2*_CH and NC*H*_*2*_CH_2_CH_2_), 2.32 (t, 2H, NCH_2_CH_2_C*H*_*2*_), 2.02 (m, H, NCH_2_C*H*_*2*_CH_2_). ^13^C‐NMR (100 MHz, DMSO‐*d*_*6*_, ẟ ppm): 168.3 (N*C*N), 142.3 (ipso aromatic carbon), 128.2, 126.9, 125.5 (aromatic carbons), 123.3 and 121.2 (N*C*H*C*HN), 72.2 (*C*HOH), 58.4 (N*C*H_2_CHOH), 48.8 (N*C*H_2_CH_2_CH_2_), 48.2 (NCH_2_CH_2_ *C*H_2_), 27.1 (NCH_2_ *C*H_2_CH_2_). Elemental Analysis: calculated for C_28_H_34_AuClN_4_Na_2_O_8_S_2_: C, 37.49; H, 3.82; N, 6.25. Found C, 37.50; H, 3.82; N, 6.24. (MALDI‐ToF, DIBAH): m/z = 861.13148 Da for [C_28_H_34_AuN_4_Na_2_O_8_S_2_]^+^; 506.53205 Da for [C_14_H_17_AuN_2_O_4_S]^+^.

(**8**) characterization ^1^H‐NMR (400 MHz, DMSO‐*d*_*6*_, ẟ ppm): 7.37–7.29 (m, 5H, aromatic protons), 6.09 (s, 1H, O*H*) 5.15 (m, 1H, C*H*OH), 4.30 (m, 4H, NC*H*_*2*_CHOH e NC*H*_*2*_CH_2_CH_2_), 2.55 (o, 2H, NCH_2_CH_2_C*H*_*2*_), 2.11 (m, 2H, NCH_2_C*H*_*2*_CH_2_). ^13^C‐NMR (100 MHz, DMSO‐*d*_*6*_, ẟ ppm): 170.0 (NCN), 141.0 (ipso aromatic carbon), 128.3, 128.1, 125.9 (aromatic carbons), 118.0 and 116.8 (N*C*Cl*C*ClN), 71.1 (*C*HOH), 56.7 (N*C*H_2_CHOH), 49.4 (NCH_2_CH_2_CH_2_), 47.8 (NCH_2_CH_2_ *C*H_2_), 26.6 (NCH_2_ *C*H_2_CH_2_). Elemental Analysis: calculated for C_28_H_30_AuCl_5_N_4_Na_2_O_8_S_2_ C, 32.50; H, 2.92; N, 5.41. Found C, 32.59; H, 3.01; N, 5.49. MALDI‐ToF (CH_3_OH, m/z): 501.03867 Da, attributable to [C_8_H_10_Cl_2_N_2_O_4_AuS]^+^ and 483.02756 Da attributable to [C_8_H_9_Cl_2_N_2_O_3_AuS]^+^.

### 2.6 Cell Culture and Treatments

Human hepatocellular carcinoma cell lines HepG2 (ATCC #HB‐8065; Manassas, VA, USA) and HuH7, kindly provided by Prof. G. Giannelli (University of Bari, Italy), were cultured in Dulbecco's Modified Eagle's Medium (Corning, New York, NY, USA), supplemented with 10% fetal bovine serum (FBS), 100 U/mL penicillin, 100 μg/mL streptomycin and 2 mM L‐glutamine (EuroClone, Milan, Italy).

The immortalized human hepatocyte (IHH) cell line, kindly provided by Prof. C. Tiribelli (Liver Research Center, Italian Liver Foundation, Trieste, Italy), was maintained in Dulbecco's Modified Eagle's Medium/Nutrient Mixture F‐12 Ham complete medium (Sigma Aldrich‐Merck, Saint Louis, MO, USA) with 10% FBS, 2 mM L‐glutamine, 100 U/mL penicillin, 100 μg/mL streptomycin, 1 μM dexamethasone and 1.2 × 10^−7^ M insulin (from bovine pancreas). All experiments were performed using cells maintained in a humidified incubator at 37°C with 5% CO_2_, and passages were limited to a maximum of 10 to preserve cell characteristics. Synthetic compounds were dissolved in Dimethyl sulfoxide (DMSO, Sigma Aldrich‐Merck, Saint Louis, MO, USA) at a 50 mM stock concentration and subsequently diluted in culture medium to achieve the desired working concentrations. Cells treated with the maximum final DMSO concentration (0.2%), corresponding to the highest vehicle concentration used to dissolve the compounds, served as vehicle controls in all experiments.

### 2.7 MTT Assay

Cytotoxic activity of our compounds was evaluated by the 3‐[4,5‐dimethylthiazol‐2‐yl]‐2,5‐diphenyltetrazolium bromide assay (MTT, Sigma Aldrich‐Merck, Saint Louis, MO, USA). Briefly, cells were seeded at a density of 1.5 × 10^4^ cells/well in a 96‐well plate, incubated for 24 h, and then treated for 24 h with increasing concentrations of 5, 6, 1, and 2 (10, 20, 30, 50, and 100 μM) or 7, 8, 3, and 4 (20, 30, 40, 50, and 100 μM). After removing the culture medium, cells were incubated with MTT solution (0.75 mg/mL) for an additional 4 h at 37°C in a cell culture incubator. The resulting water‐insoluble formazan crystals were dissolved in a 1:1 isopropanol: DMSO solution containing 1% Triton X‐100 (Sigma Aldrich‐Merck, Saint Louis, MO, USA) and absorbance was measured at 570 nm (with background subtraction at 630 nm) using a Multiskan Go spectrophotometer (Thermo Scientific). For HepG2 cells, the MTT assay was also performed by adding 10 mM N‐acetyl‐L‐cysteine (NAC, Sigma Aldrich‐Merck, Saint Louis, MO, USA) 1 h before each treatment. Cell viability is expressed as the percentage of viable cells relative to cells treated with 0.2% DMSO, which were arbitrarily set at 100%. The half‐maximal inhibitory concentration (IC50), with 95% confidence intervals (95% CI), was determined using nonlinear regression analysis with GraphPad Prism software (version 8.4.2, GraphPad Software, San Diego, CA, USA).

### 2.8 Cell Morphological Evaluation

HepG2 cells were seeded at a density of 2 × 10^5^ cells/well in a 12‐well plate and treated for 24 h with increasing concentrations of 7 (20, 30, and 40 μM) or 8 (30, 40, and 50 μM). Cell morphological alterations were examined using an inverted phase‐contrast microscope (Nikon Eclipse TS100, Tokyo, Japan; 40× magnification), while nuclear morphology was assessed through Hoechst 33258 staining, as previously described (Armentano et al. 2015), and stained cells were analyzed using the FLoid Cell Imaging Station (Thermo Fisher Scientific). The negative control was represented by cells treated with the maximum DMSO concentration used.

### 2.9 Intracellular ROS Detection

The intracellular levels of reactive oxygen species (ROS) were measured using 2′,7′‐dichlorofluorescein diacetate (DCFH‐DA, Sigma Aldrich‐Merck, Saint Louis, MO, USA), a cell‐permeable fluorogenic probe. Briefly, HepG2 cells were seeded in a 24‐well plate (1 × 10^5^ cells/well) and treated with 7 (20, 30, and 40 μM) or 8 (30, 40, and 50 μM) for 24 h. Cells were then incubated with 1 μM DCFH‐DA for 1 h at 37°C in a humidified 5% CO_2_ atmosphere. After incubation, cells were detached with Trypsin–EDTA Solution (Sigma Aldrich‐Merck, Saint Louis, MO, USA), washed with Dulbecco's Phosphate Buffered Saline (PBS, EuroClone, Milan, Italy), and analyzed using FACScan flow cytometry (BD Biosciences) with an excitation wavelength of 488 nm and an emission wavelength of 530 nm. Cells treated with 1 mM tert‐butyl hydroperoxide (TBHP) served as a positive control for ROS generation. The experiment was also performed by pre‐treating cells with 10 mM NAC for 1 h prior to the assay.

### 2.10 Western Blot Analysis

HepG2 cells were grown in 6‐well plates (5 × 10^5^ cells per well), treated with increasing concentrations of 7 (20, 30, and 40 μM) or 8 (30, 40, and 50 μM) for 24 h, and subsequently lysed using RIPA buffer (Cell Signaling Technology, CST, Danvers, MA, USA) supplemented with a protease inhibitor cocktail. Protein concentration was determined using the Bradford assay (Sigma Aldrich‐Merck, Saint Louis, MO, USA) (Bradford 1976) and equal amounts of protein samples were separated by SDS‐PAGE and transferred onto nitrocellulose membranes. Following 1 h of incubation at room temperature in blocking buffer (5% *w*/*v* non‐fat dry milk in TRIS‐buffered saline, pH 7.4, containing 0.1% (*v*/*v*) Tween‐20 (TBST)), membranes were incubated overnight at 4°C with specific primary antibodies diluted in blocking buffer: anti‐poly‐ADP‐ribose polymerase (PARP, 1:1000), anti‐Caspase‐3 (1:1000), anti mTOR (1:5000), anti p‐mTOR (1:2000), anti AKT (1:1000), anti p‐AKT (1:2000), anti‐Beclin‐1 (1:1000), anti‐LC3 A/B (1:1000) and anti‐β‐actin (1:1000).

Primary antibodies specific for anti‐PARP (#9542), LC3A/B (#12741), β‐actin (#3700), AKT (#4691), p‐AKT (Ser473) (#4060), were purchased from Cell Signaling Technology (CST, Danvers, MA, USA). Primary antibodies specific for Beclin‐1 (#849701), was purchased from Biolegend (San Diego, CA, USA). Primary antibodies specific for m‐TOR (#66888‐1‐Ig), p‐mTOR (Ser2448) (#67778‐1‐Ig), Caspase‐3 (#66470‐2‐Ig), were purchased from Proteintech (San Diego, CA, USA).

Each membrane was then washed three times with TBST and incubated with the appropriate HRP‐conjugated secondary antibody for 1 h at room temperature (antimouse IgG‐HRP‐linked (#7076), and anti‐rabbit IgG‐HRP‐linked (#7074), Cell Signaling Technology, Danvers, MA, USA). After three additional washes with TBST, immunoreactive bands were detected using enhanced chemiluminescence reagents (ECL Star Enhanced Chemiluminescent Substrate, LiteAblot TURBO Extra Sensitive Chemiluminescent Substrate, EuroClone, Milan, Italy) and ChemiDoc XRS detection system with ImageLab software (Bio‐Rad, Hercules, CA, USA). The relative intensity of bands was performed by ImageJ software (version 1.52a, National Institutes of Health, Bethesda, MD, USA), normalized to β‐actin and represented as fold change relative to the control group (DMSO‐treated cells).

### 2.11 Statistical Analysis

Data, obtained from three independent experiments performed in triplicate, were normalized to their respective DMSO‐treated controls and presented as means ± standard error measurement (SEM). Significant differences between means were analyzed by one‐way analysis of variance (ANOVA) followed by Dunnett's post hoc test, using GraphPad Prism software (version 8.4.2, GraphPad Software, San Diego, CA, USA). Results were considered statistically significant for *p*‐values < 0.05.

## 3 Results

### 3.1 Synthesis of the Metal Complexes

**SCHEME 1:**
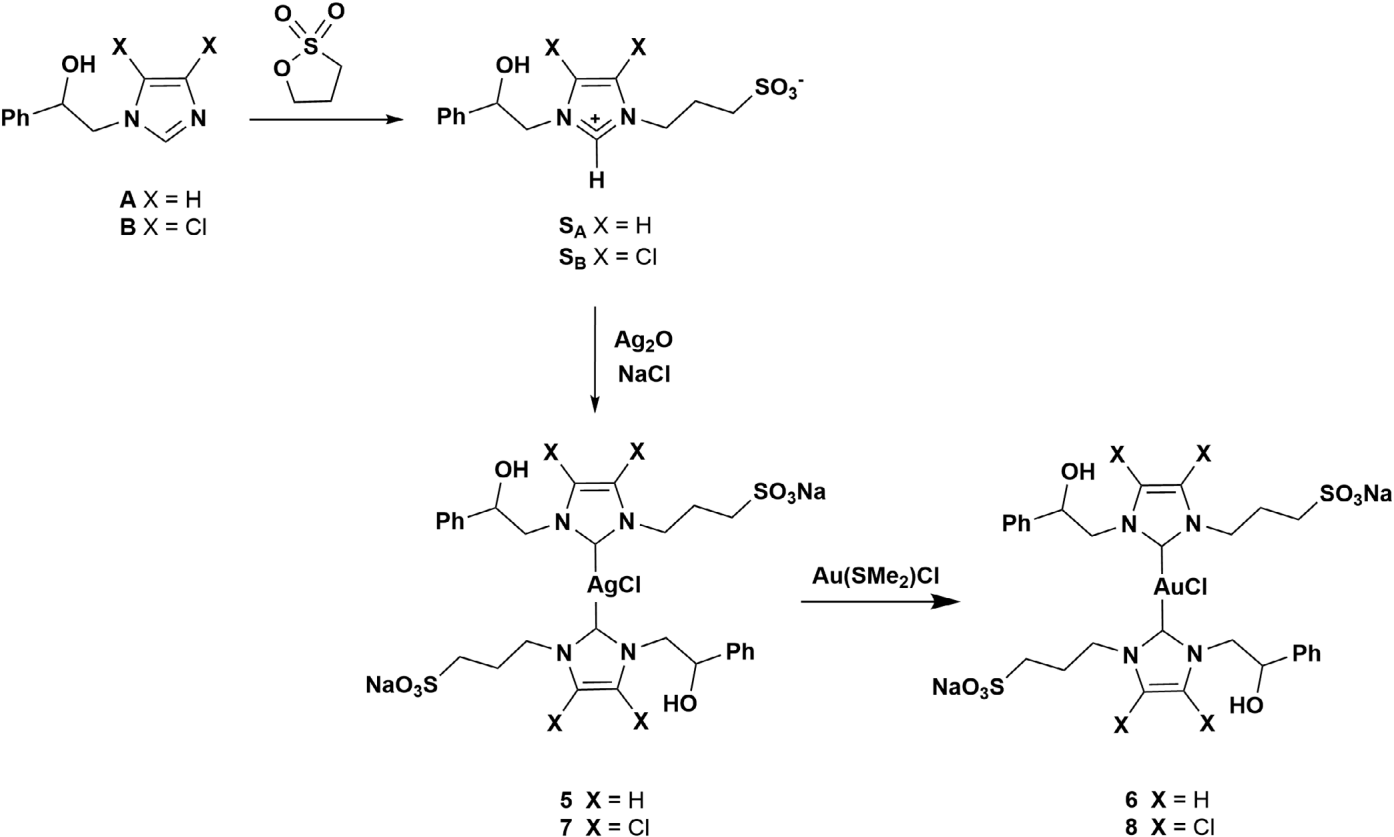
Synthetic pathways of sulfonated Ag(I) and Au(I) NHC complexes.

The new sulfonated NHC complexes were synthesized following the synthetic procedure illustrated in Scheme 1.

The new silver‐based complexes **5** and **7** were prepared through three reaction steps, while the gold compounds **6** and **8** were obtained by trans‐metalation using the silver‐NHC precursor. A and B compounds have been synthesized using the synthetic procedure already reported by some of us (D'Amato et al. 2024; Mariconda et al. 2020; Napoli et al. 2013). The NMR analyses give the same data reported in the literature. The sulfonate‐functionalized‐NHCs were obtained by synthesizing the most appropriate precursors, such as the corresponding imidazolium derivatives (S_A_ and S_B_): their deprotonation generates the analogous carbenes in situ. S_A_ and S_B_ were obtained by reacting the compounds A and B with 1,3‐propane sultone as an alkylating agent in acetonitrile at reflux. The two reactions were conducted at different times: 24 h for A and 48 h for B. Both reaction products were recovered by forcing precipitation by adding acetone with a yield of about 90% and 50%, respectively, in racemic form. S_A_ and S_B_ imidazolium salts are zwitterionic racemic molecules, with the cationic charge internally balanced by one sulfonate. Spectroscopic (^1^H and ^13^C{^1^H} NMR) and analytical data (elemental analysis and mass spectrometry) were in complete agreement with the proposed structures (details are given in the Experimental Section). For both desired products, their formation is clear in the ^1^H and ^13^C spectra for the appearance of signals, at high fields, of the methylene protons of the added three‐term chain and its carbons. Diagnostic are the signals related to cationic carbon and the proton bound to it. In ^13^C NMR spectra, methine carbons are found at 137.0 ppm for S_A_ and 140.4 ppm for S_B_, respectively, while in ^1^H NMR spectra the proton bound to them is found at about 9 ppm for both imidazolium salts. The MALDI‐TOF spectra of S_a_ e S_b_, carried out in methanol, supported the proposed structures. As depicted in Scheme 1, the general preparation of complexes **5** and **7** implied the reaction between Ag_2_O and the corresponding imidazolium salt in refluxing methanol. For this purpose, complexes **5** and **7** were obtained by slightly modifying the procedure reported by Flores and Jesus (Baquero et al. 2013), where NaCl is added to the imidazolium salt and the metal precursor. As reported (Baquero et al. 2013), the addition of NaCl provides the formation of bis‐carbene complexes as the intermediate species Ag[Ag(NHC)_2_], containing a naked Ag^+^ counterion, evolves to the desired complexes after replacement with Na^+^. Sodium bis‐carbene silver complexes exhibit stability in both their solid form and in solution, even under prolonged exposure to light. The Ag and Au complexes were characterized using ^1^H‐, ^13^C‐NMR, elemental analysis, and mass spectroscopy. For the NHC metal complexes **5** and **7**, the imidazolium proton (NC*H*N) resonances disappeared in the ^1^H‐NMR spectrum, though the chemical shifts for other protons mirrored those of the corresponding precursors. In ^13^C‐NMR spectra, carbene carbons appear as doublets because Ag is present for 52% as ^107^Ag and 48% as ^109^Ag with spin ½. The corresponding NHC‐Au(I) complexes **6** and **8** were then synthesized through a silver‐carbene transfer method using (SMe_2_)AuCl as a reagent in methanol. The characterization of gold complexes **6** and **8** confirms the success of the synthesis of gold analogues; in fact, in the ^13^C spectra of **6** and **8**, complexes are evident, the carbene carbons at 168.3 ppm for complex **6** and at 163.9 ppm for complex **8** confirmed the coordination of the imidazol‐2‐ylidene group to gold. All complexes were also characterized by MALDI‐TOF mass spectrometry in methanol, confirming the hypothesized structures.

### 3.2 Silver and Gold Carbene Complexes Reduce Hepatoma Cell Lines Viability

**FIGURE 3:**
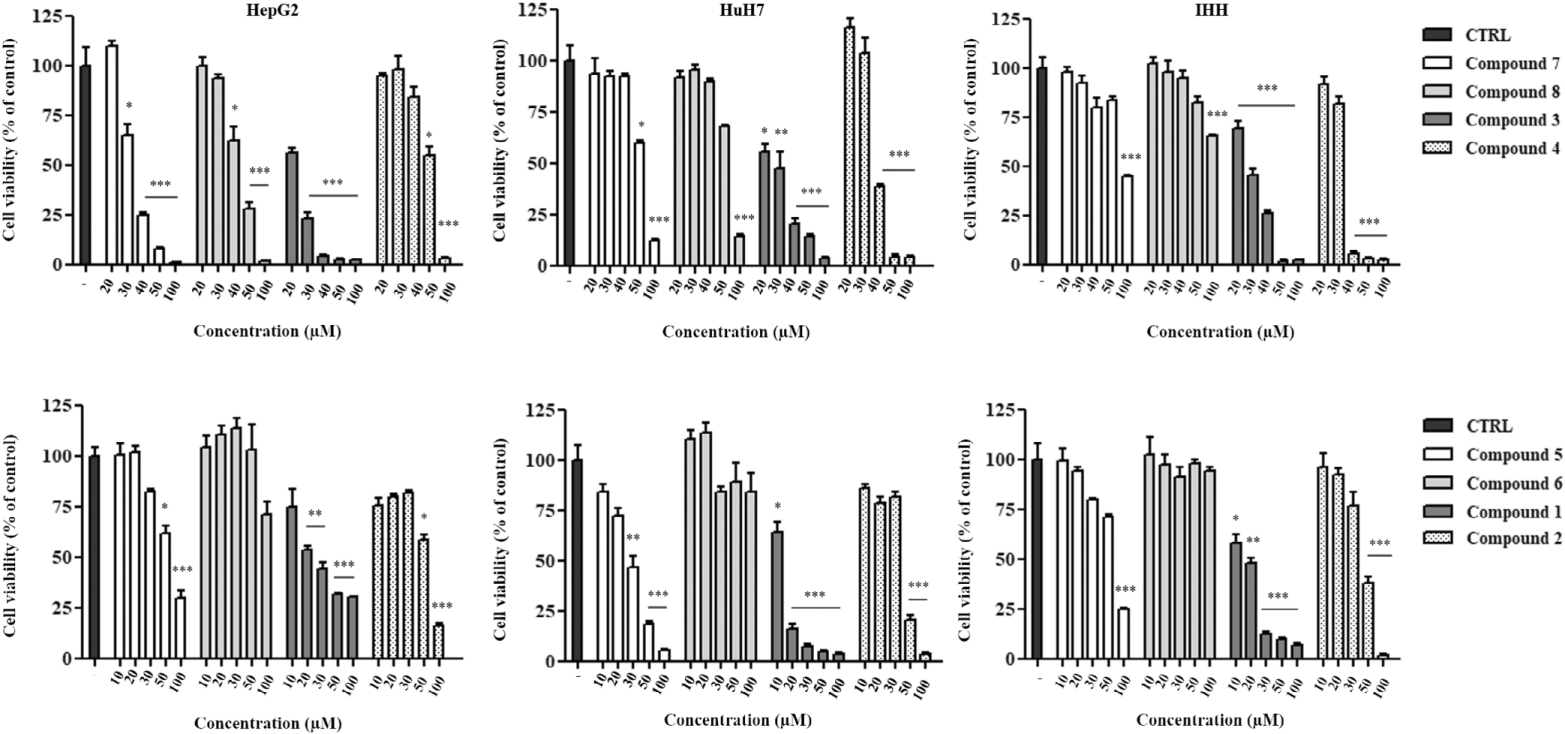
Cytotoxic effect of silver and gold carbene complexes on hepatoma cell lines (HepG2 HuH7) and normal hepatocytes (IHH), evaluated by MTT assay. Cells were exposed for 24 h to indicated concentrations of different complexes and the relative cell viability was calculated as the percentage respect to cells treated with vehicle (indicated with minus sign or with CTRL abbreviation). Data are shown as means ± SEM of three experiments, each performed in triplicate. Significant differences were evaluated by one‐way ANOVA followed by Dunnett's post hoc test (*p < 0.05, **p < 0.01, ***p < 0.001), using GraphPad Prism 8.4.2 software.

We first evaluated the cytotoxicity of several silver and gold carbene complexes on two hepatocarcinoma cell lines with different degrees of differentiation (HepG2 and HuH7) and on a non‐malignant liver cell line (IHH) using the MTT assay. Cells were treated with increasing concentrations (10, 20, 30, 50, and 100 μM or 20, 30, 40, 50, and 100 μM) of different compounds (**1–8**) for 24 and 48 h. Cells treated with DMSO were used as a negative control. As shown in Figure 3 and Table 1, all compounds reduced cell viability in a dose‐dependent manner. Only the **6** complex exhibited low cytotoxicity against both cancerous and healthy cells (IC50 > 100 μM). However, no significant reduction in IC50 was observed between 24 and 48 h of treatment, suggesting that the main cytotoxic effect of the synthesized compounds occurs within the first hours of exposure (Table 1). Among the tested compounds:

- **3** strongly reduced cell viability, but no significant differences were observed between the three cell lines.
- **4**, **1**, **2**, and **5** exhibited lower cytotoxicity in one of the two cancer cell lines compared to the healthy cell line.
- **7** and **8** displayed significantly higher cytotoxicity against HepG2 and HuH7 cells than non‐tumor cells, encouraging further investigations.

**TABLE 1:** The half maximal inhibitory concentration (IC50) values and their 95% confidence intervals (95% CI) were determined by nonlinear regression analysis using GraphPad Prism software (version 8.4.2, GraphPad Software, San Diego, California, USA).

Complex	IC50 (95% CI) μM 24 h			IC50 (95% CI) μM 48 h		
	IHH	HepG2	HuH7	IHH	HepG2	HuH7
**1**	13.62 (11.02–14.89)	24.48 (15.71–25.79)	12.05 (11.26–12.88)	12.15 (11.37–12.99)	14.88 (14.19–16.05)	3.848 (2.235–6.625)
**2**	39.73 (30.94–41.62)	53.04 (50.48–58.01)	37.14 (32.89–41.94)	30.77 (26.89–35.20)	65.58 (52.44–67.17)	31.90 (29.94–34.00)
**3**	29.31 (19.63–43.78)	21.39 (20.79–22.02)	23.85 (20.32–26.60)	14.06 (13.56–14.58)	12.79 (12.22–13.39)	15.08 (14.19–16.05)
**4**	32.53 (29.07–36.41)	54.11 (50.48–58.01)	38.87 (34.25–41.98)	28.17 (26.75–29.67)	59.35 (52.44–67.17)	35.05 (32.31–34.09)
**5**	64.01 (58.22–70.37)	74.43 (62.99–80.12)	28.06 (26.27–29.98)	56.46 (49.61–64.26)	54.42 (44.68–66.28)	21.98 (20.51–23.56)
**6**	> 100	> 100	> 100	> 100	> 100	> 100
**7**	95.01 (85.85–105.2)	31.51 (30.87–32.19)	52.14 (49.35–55.09)	40.30 (35.34–45.95)	27.89 (26.78–29.05)	36.15 (34.25–41.98)
**8**	> 100	43.84 (42.40–44.73)	63.60 (55.21–68.47)	68.67 (58.34–80.82)	43.09 (41.92–44.29)	57.9 (52.44–67.17)

Since the lowest IC50 value was recorded in HepG2 cells, this cell line was selected for subsequent experiments. Furthermore, considering that the IC50 values at 24 h for **7** and **8** were 31.51 and 43.84 μM, respectively, the following concentrations were selected for dose‐dependent assays: **7**: 20, 30, and 40 μM and **8**: 30, 40, and 50 μM.

### 3.3 Complexes 7 and 8 Induce Morphological Alterations in HepG2 Cells

**FIGURE 4:**
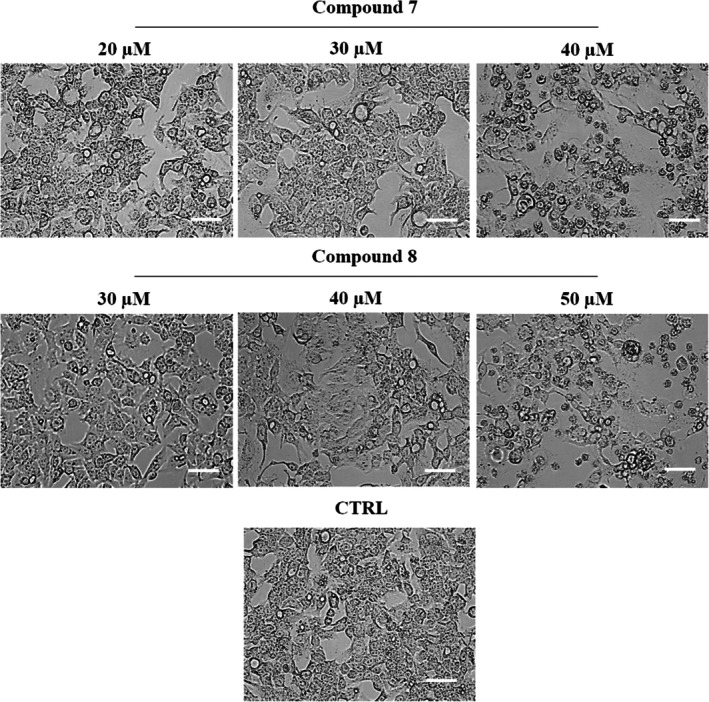
Morphological evaluation of HepG2 cells following treatment with carbene complexes at the indicated concentrations. Cells were observed using inverted microscopy (40× magnification). Scale bars: 100 μm. The images shown are representative of three independent experiments.

To investigate the cytotoxic effects of **7** and **8** complexes, HepG2 cells were treated for 24 h with three different concentrations of both compounds, as shown in Figure 4, and cell morphology was analyzed using a light optical microscope (Nikon Eclipse TS100, 40× magnification). No significant differences were observed in the number or morphology of HepG2 cells exposed to **7** (20 μM) or **8** (30 μM), in accordance with the MTT assay results. In contrast, cells treated with both complexes at their IC50 concentrations exhibited a higher number of intracellular vacuoles. Furthermore, treatment with the highest concentrations of both compounds led to marked morphological changes, including a dramatic increase in intracellular vacuoles and a greater number of detached, round‐shaped cells, clearly indicating a dose‐dependent cytotoxic effect.

### 3.4 Treatment With 7 or 8 Induces Non‐Apoptotic Cell Death in HepG2 Cells

**FIGURE 5:**
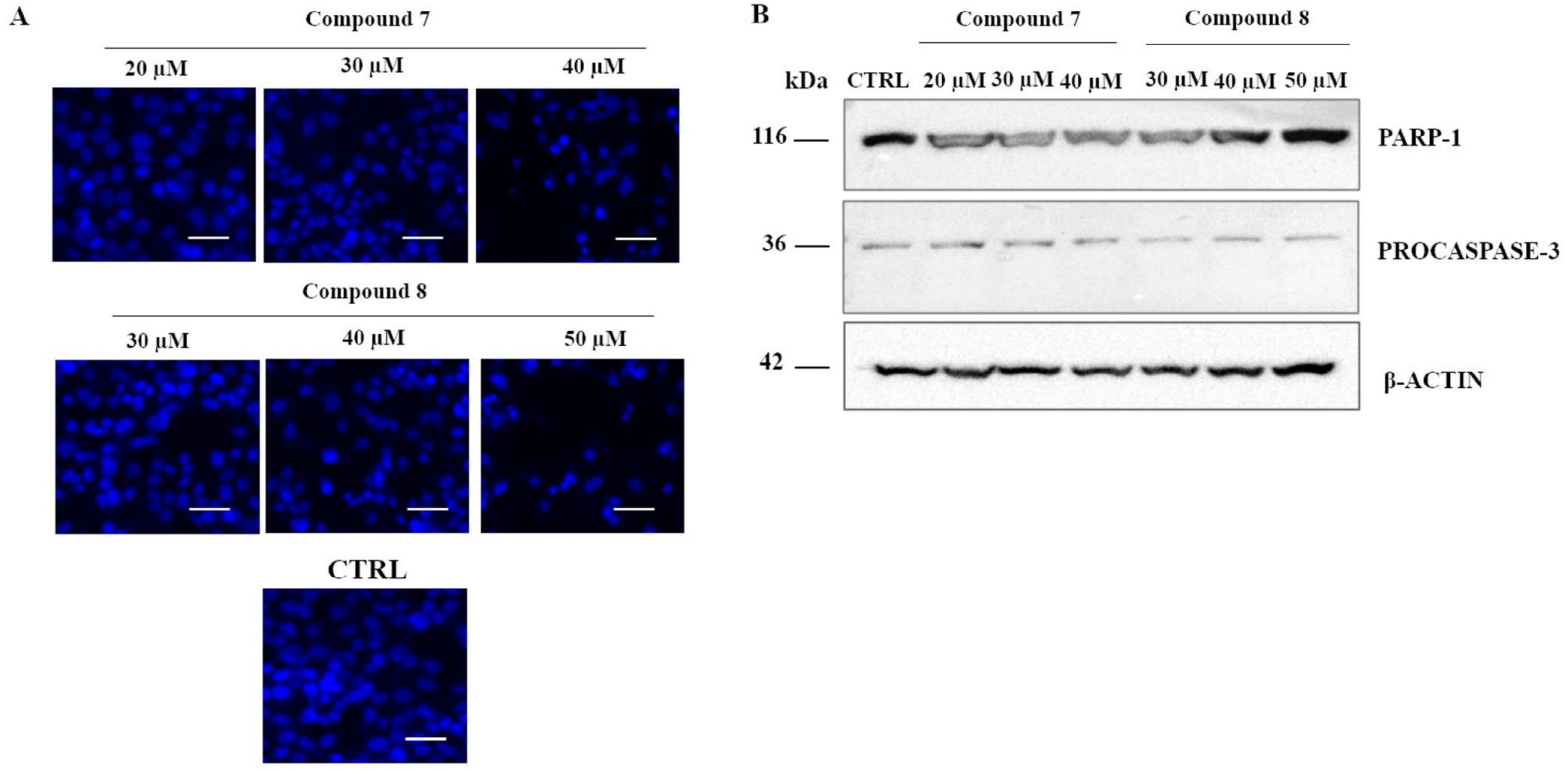
Apoptosis assays of HepG2 cells treated with carbene complexes for 24 h. (A) Representative fluorescence images of Hoechst 33258 stained HepG2 cells. Scale bar: 100 μm. (B) Western blot analysis of HepG2 cells treated with our compounds. β‐Actin was used as the loading control.

To determine whether HepG2 cells treated with **7** or **8** complexes undergo apoptosis, we assessed nuclear changes using Hoechst 33342 staining and examined caspase‐3 activation and PARP‐1 cleavage via Western blot analysis. As shown in Figure 5A, the nuclear structure of HepG2 cells treated with both complexes remained intact, with no evidence of chromatin condensation or apoptotic body formation. Western blot analysis did not detect the active 17 kDa subunit of caspase‐3 or the cleaved PARP‐1 form, clearly indicating that caspase‐3 was not activated by these compounds (Figure 5B). These results demonstrate that our compounds are able to activate non‐apoptotic cell death pathways.

### 3.5 Complexes 7 and 8 Induce Autophagy via the AKT/mTOR Signaling Pathway

**FIGURE 6:**
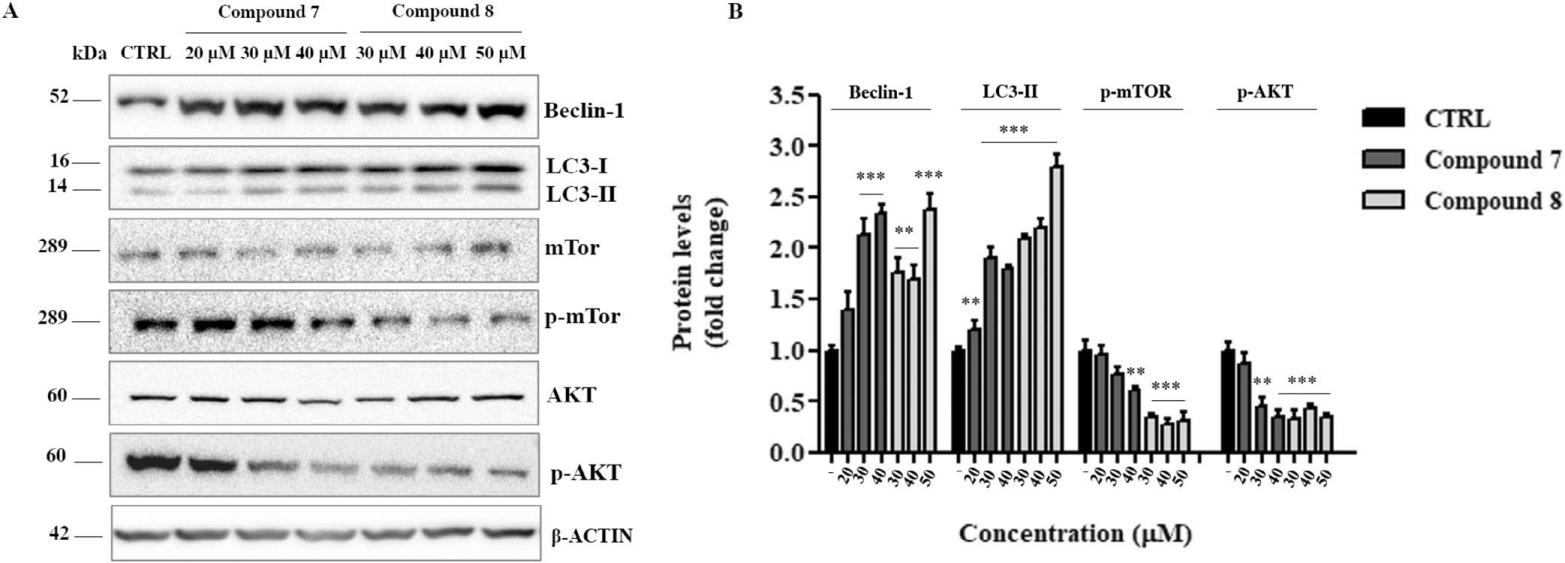
Autophagy induction in HepG2 cells treated with 7 or 8, evaluated by Western blot analysis. (A) Representative Western blot images and (B) densitometric analysis of HepG2 cells exposed to the indicated concentrations of both complexes for 24 h. β‐actin was used as the loading control. All data are expressed as mean ± standard error (SE) from three independent experiments and shown as fold change relative to the control group (DMSO‐treated cells). Significant differences were evaluated by one‐way ANOVA followed by Dunnett's post hoc test (*p < 0.05, **p < 0.01, ***p < 0.001), using GraphPad Prism 8.4.2 software.

To investigate whether autophagy was activated by our compounds, we analyzed by immunoblotting the expression of two key autophagy biomarkers: Beclin‐1, crucial in the early stages of autophagy, and microtubule‐associated protein 1 light‐chain 3 (LC3)‐II, generated by the conjugation of cytosolic LC3‐I to phosphatidylethanolamine (PE) and localized on the surface of the nascent autophagosome. (Parzych and Klionsky 2014). As shown in Figure 6, compared to the control group, the protein expression levels of Beclin‐1 and LC3‐II significantly increased in a dose‐dependent manner in HepG2 cells exposed to **7** or **8** for 24 h. To further explore the mechanism of autophagy induction, we examined the levels of phosphorylated mTOR, a major negative regulator of autophagy, which inhibits the early steps of the process. Compared to the negative control, the levels of phosphorylated mTOR decreased in HepG2 cells treated with both compounds in a dose‐dependent manner, while the total mTOR levels remained unchanged, suggesting that mTOR activity was suppressed (Figure 6). Since mTOR activity is regulated by AKT signaling, where AKT suppression leads to decreased mTOR activity and enhanced autophagy, we also analyzed phosphorylated AKT levels. Consistent with the changes observed in mTOR, the levels of phosphorylated AKT decreased in HepG2 cells treated with **7** and **8**, while the total AKT levels remained unchanged (Figure 6). Overall, these findings suggest that the AKT/mTOR signaling pathway is involved in the autophagy induced by our compounds.

### 3.6 Gold and Silver Carbene Complexes Increase Intracellular ROS Level in HepG2 Cells

**FIGURE 7:**
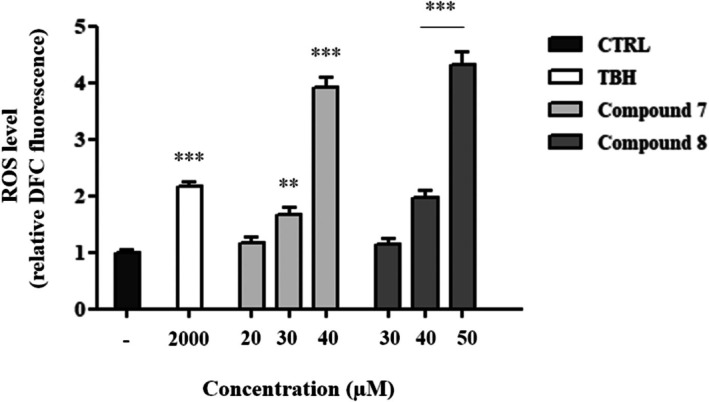
Flow cytometry analysis of intracellular ROS levels in HepG2 cells treated with gold and silver carbene complexes for 4 h. Data are presented as mean ± SEM from three independent experiments, each performed in triplicate. Statistical significance was assessed using one‐way ANOVA followed by Dunnett's post hoc test (**p < 0.01, *p < 0.001), using GraphPad Prism 8.4.2 software.

Inhibition of the mammalian target of rapamycin (mTOR) signaling pathway and the subsequent autophagic process can be triggered by the generation of reactive oxygen species (ROS). To assess this, we measured ROS accumulation in HepG2 cells treated with three different concentrations of both compounds for 4 h, by detecting the increase in 2′,7′‐dichlorofluorescein (DCF) fluorescence. Cells exposed to 1 mM tert‐butyl hydroperoxide (TBHP), a well‐established pro‐oxidant agent widely used to induce intracellular ROS in vitro models (Figueroa et al. 2018; García‐Pérez et al. 2021; Soragni et al. 2022), served as a positive control. The lowest concentration of both compounds did not lead to a significant increase in ROS production. In contrast, ROS levels increased approximately twofold following exposure to 30 μM of compound **7** or 40 μM of compound **8**, and up to fourfold at the highest concentration used for both compounds (Figure 7). These results indicate that our complexes influence ROS production in a dose‐dependent manner. Since mitochondria are a major source of ROS, we evaluated mitochondrial membrane potential (ΔΨm), a global indicator of mitochondrial function, by measuring the relative fluorescence of tetramethylrhodamine methyl ester (TMRM). No significant changes in ΔΨm were observed in cells treated with either compound (data not shown), suggesting that ROS were generated from other cellular compartments.

### 3.7 Compounds 7 and 8 Induce Autophagy‐Mediated Cell Death via ROS Production

**FIGURE 8:**
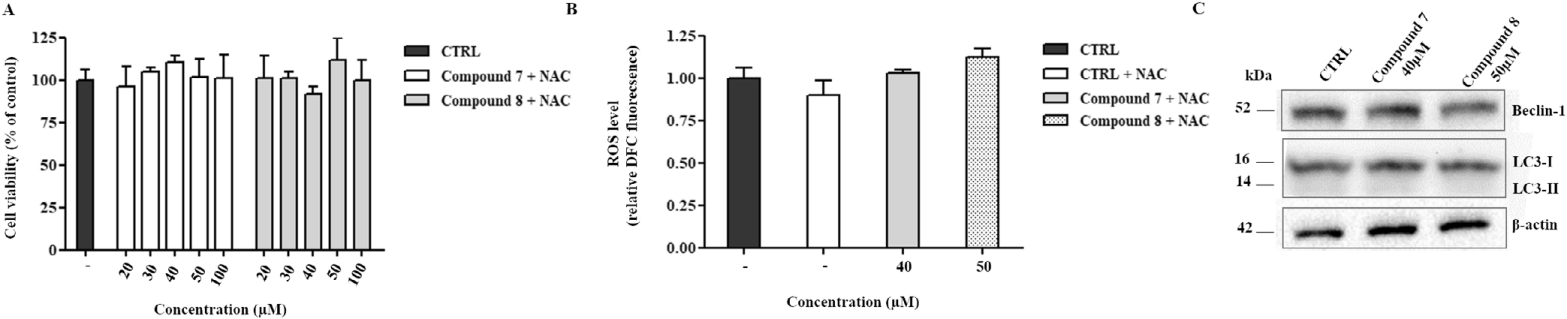
Cell viability and autophagy analysis in HepG2 cells treated with gold and silver carbene complexes following ROS inhibition. (A) MTT assay performed on HepG2 cells pre‐treated with NAC for 1 h, followed by 24‐h exposure to 7 or 8 at the indicated concentrations. Results are expressed as the percentage of viable cells relative to the control group (indicated with a minus sign or the abbreviation CTRL). (B) Intracellular ROS levels were measured in HepG2 cells pre‐treated with NAC for 1 h, then exposed for 4 h to each complex at its IC50 concentration. (C) Western blot analysis of HepG2 cells pre‐treated with NAC for 1 h, followed by 24‐h incubation with **7** or **8** at their IC50 concentrations.

To determine whether cell death induced by our compounds is ROS‐dependent, cell viability was assessed in HepG2 cells pre‐treated with 10 mM N‐acetyl‐L‐cysteine (NAC), a free radical scavenger, for 1 h before the administration of **7** and **8** complexes. As shown in Figure 8A, NAC pre‐treatment provided complete protection against the cytotoxic effects of both complexes, suggesting that ROS production acts as a key trigger for cell death induced by our compounds. Since several studies have reported that autophagy is activated in response to oxidative stress, we further investigated this mechanism by treating HepG2 cells with the IC50 concentration of both carbene complexes. ROS production was analyzed after 4 h, while the expression levels of Beclin‐1 and LC3‐II were assessed after 24 h. As shown in Figure 8B,C, in NAC‐treated HepG2 cells, we did not observe an increase in intracellular ROS levels nor an increase in Beclin‐1 and LC3‐II expression, indicating that autophagy induction by our compounds is ROS‐mediated.

## 4 Discussion

Hepatocellular carcinoma is one of most common and aggressive malignant cancers worldwide Despite advances in conventional chemotherapy, molecular targeted therapy, and immunotherapy (Hamaya et al. 2023), whether as monotherapies or in combinatorial strategies (Guardascione and Toffoli 2020), treatments for advanced HCC still demonstrate limited efficacy, largely due to the development of multidrug resistance (MDR). MDR is a complex tumor phenotype characterized by the cross‐resistance of cancer cells to a broad spectrum of structurally unrelated cytotoxic agents (Duan et al. 2019). A key factor contributing to MDR is the evasion of apoptosis, the most extensively studied programmed cell death mechanism and a well‐recognized hallmark of cancer (Hanahan and Weinberg 2011; Neophytou et al. 2021; Reddi and Kumar 2004). For this reason, while apoptosis induction was long considered the primary goal of cancer therapy, current research is increasingly focused on developing novel anticancer agents capable of triggering alternative cell death forms to overcome apoptosis resistance (Balaji et al. 2021; Wang et al. 2022). Moreover, there is an urgent need for new therapeutic agents with high antitumor efficacy and reduced toxicity toward healthy cells, aiming to minimize adverse side effects and improve treatment outcomes. Since the discovery of the antineoplastic activity of cisplatin, platinum‐based drugs have been widely used, both as monotherapies and in combination regimens, for treating various solid tumors, including HCC. These compounds exert anticancer effects mainly by binding to nuclear DNA, thereby interfering with transcription and DNA replication. However, their lack of selectivity results in toxicity to both malignant and normal cells, leading to severe side effects and off‐target organ damage (Hamaya et al. 2023; Zhang et al. 2022). To mitigate these limitations, numerous transition metal‐based complexes have been synthesized over the past decades, with their antitumor activity evaluated against various cancer cell lines (Lucaciu et al. 2022; Prathima et al. 2023). Among non‐platinum metal complexes, we focused on silver(I) and gold(I) N‐heterocyclic carbene (NHC) complexes, which have shown promising chemotherapeutic potential, by exerting strong cytotoxic effects through multiple mechanisms, including DNA binding and cleavage, enzyme inhibition, ROS generation, and apoptosis induction (Augello et al. 2023; Kumar Raju et al. 2022; Lu et al. 2022). In this study, we evaluated the antitumor potential of several silver(I) and gold(I) NHC complexes in two liver cancer cell lines with different degrees of differentiation (HepG2 and HuH7) and in the immortalized non‐cancerous liver cell line (IHH). Among the tested compounds, complexes **7** and **8** exhibited the highest dose‐dependent cytotoxicity against both HCC cell lines, while displaying lower toxicity toward non‐malignant cells. We therefore selected HepG2 cells for further investigations, as they showed higher sensitivity to both compounds (IC50: 31.51 μM for **7** and 43.84 μM for **8**). Morphological analysis revealed that both compounds induced significant dose‐dependent changes, characterized by intracellular vacuolization and round‐shaped cells. Unlike other silver and gold complexes, our molecules did not trigger apoptosis, as evidenced by the absence of chromatin condensation and PARP‐1 cleavage. Instead, they induced autophagy, as confirmed by the up‐regulation of Beclin‐1, a key regulator of early autophagosome formation, and LC3‐II, the conjugated form of cytosolic LC3‐I, which is stably bound to autophagosomal membranes (Parzych and Klionsky 2014). Autophagy is a highly conserved cellular process responsible for protein and organelle degradation, involving the formation of double‐membrane vesicles (autophagosomes), that contribute to cellular homeostasis (Gómez‐Virgilio et al. 2022). While autophagy primarily serves as a cytoprotective response under stress conditions (e.g., nutrient deprivation, oxidative stress, hypoxia), it can also lead to a non‐apoptotic programmed cell death, known as type II programmed cell death (Liu et al. 2023). In cancer progression, autophagy plays a dual role, acting as either a tumor‐promoting or tumor‐suppressing mechanism, depending on the cellular context and tumor stage. On one hand, autophagy can drive tumorigenesis by helping cancer cells adapt to stressful conditions, such as hypoxia and immune responses, thereby contributing to drug resistance. On the other hand, autophagy can function as a tumor suppressor by triggering cell death, thereby enhancing the efficacy of anticancer drugs (Folkerts et al. 2019; Kwantwi 2024; Singh et al. 2018). In cases where autophagy promotes tumor survival, autophagy inhibitors can be employed to sensitize cancer cells to therapy and enhance the cytotoxic effects of chemotherapeutic agents (Wang et al. 2016; Zhang et al. 2022). Conversely, when autophagy induces cancer cell death, autophagy‐promoting drugs, including various natural compounds and therapeutic agents, could provide an effective strategy for targeting apoptosis‐resistant tumors (Chen et al. 2024; Jiang et al. 2017; Ma et al. 2024). The AKT/mTOR signaling pathway is a major regulator of autophagy (Li et al. 2017; Xu et al. 2020). Our results demonstrate that compounds **7** and **8** significantly reduce AKT and mTOR phosphorylation, suggesting that their cytotoxic effects are linked to AKT/mTOR pathway suppression and autophagy induction. mTOR, modulated by the PI3K/AKT survival pathway, plays a crucial role in cancer progression by promoting proliferation, invasion, metastasis, and drug resistance, ultimately contributing to HCC progression (Ahmadi‐Dehlaghi et al. 2023; Cui et al. 2020; Tian et al. 2023). Therefore, an effective strategy for cancer therapy could target the PI3K/AKT/mTOR pathway. Rapamycin and its derivatives (Temsirolimus, Everolimus, and Ridaforolimus) are well‐established mTOR inhibitors currently used as immunosuppressants and chemotherapeutic agents (Wang et al. 2025).

Several studies have shown that excessive ROS production can also induce autophagy via AKT/mTOR pathway inhibition (Makhov et al. 2014; Ni et al. 2021). ROS, generated by several cellular processes, represent highly reactive oxygen species, capable of oxidizing cellular components, leading to structural and functional damage (Ghosh et al. 2018; Juan et al. 2021). Such oxidative stress can ultimately lead to various forms of cell death, including apoptosis and autophagy. Tumor cells, compared to normal cells, exhibit elevated basal ROS levels, making them more susceptible to ROS‐inducing pharmacological treatments (Redza‐Dutordoir and Averill‐Bates 2016; Su et al. 2019). To explore this mechanism, we assessed ROS levels in HepG2 cells treated with compounds **7** and **8**. Both induced dose‐dependent ROS accumulation, which was completely abolished by pre‐treatment with NAC, preventing both cytotoxicity and autophagy.

Our results demonstrated that silver(I) and gold(I) NHC complexes **7** and **8** are capable of inducing ROS‐dependent autophagic cell death in a hepatoma cell line, inhibiting the AKT/mTOR pathway. The growing evidence linking autophagy and cancer suggests that targeted manipulation of this process could represent an innovative and promising therapeutic strategy. These results warrant further in vivo investigations to explore the full potential of these compounds in apoptosis‐resistant cancers.

## 5 Conclusion

The in vitro cytotoxic potential of eight silver and gold metal complexes (**1**–**8**) was evaluated in HepG2 and HuH7 hepatocarcinoma cell lines. Four of these complexes were newly synthesized, incorporating a sulfonate group to enhance water solubility. The metal complexes were characterized by ^1^H and ^13^C NMR, elemental analysis, and mass spectrometry. Our findings demonstrate that complexes **7** and **8** induce autophagic cell death through the AKT/mTOR pathway inhibition, mediated by ROS generation. These results highlight their potential as promising candidates for overcoming apoptosis resistance in HCC therapy. Further in vivo studies are warranted to validate their therapeutic efficacy and elucidate their mechanisms of action in greater detail.

## Author Contributions

**Faustino Bisaccia:** writing – review and editing, conceptualization, supervision. **Maria Francesca Armentano:** conceptualization, data curation, writing – original draft, supervision. **Francesco Viceconte:** investigation, methodology, formal analysis. **Rocchina Miglionico:** formal analysis, writing – original draft, investigation, visualization. **Annaluisa Mariconda:** conceptualization, data curation, writing – original draft, supervision, visualization. **Pasquale Longo:** writing – review and editing, conceptualization, supervision.

## Funding

The authors have nothing to report.

## Conflicts of Interest

The authors declare no conflicts of interest.

## Supporting information


**Figure S1:** cbdd70283‐sup‐0001‐FigureS1‐S16.doc. ^1^H‐NMR spectrum of compound A (400 MHz, DMSO‐d_6_).
**Figure S2:** 13C‐NMR spectrum of compound A (75 MHz, DMSO‐d6).
**Figure S3:** 1H‐NMR spectrum of compound SA (400 MHz, DMSO‐d6).
**Figure S4:** 13C‐NMR spectrum of compound SA (100 MHz, DMSO‐d6).
**Figure S5:** 1H‐NMR spectrum of complex 5 (400 MHz, DMSO‐d6).
**Figure S6:** 13C‐NMR spectrum of complex 5 (100 MHz, DMSO‐d6).
**Figure S7:** 1H‐NMR spectrum of complex 6 (400 MHz, DMSO‐d6).
**Figure S8:** 13C‐NMR spectrum of complex 6 (100 MHz, DMSO‐d6).
**Figure S9:** 1H‐NMR spectrum of compound B (300 MHz, DMSO‐d6).
**Figure S10:** 13C‐NMR spectrum of compound B (75 MHz, DMSO‐d6).
**Figure S11:** 1H‐NMR spectrum of compound SB (300 MHz, DMSO‐d6).
**Figure S12:** 13C‐NMR spectrum of compound SB (100 MHz, DMSO‐d6).
**Figure S13:** 1H‐NMR spectrum of complex 7 (400 MHz, DMSO‐d6).
**Figure S14:** 13C‐NMR spectrum of complex 7 (100 MHz, DMSO‐d6).
**Figure S15:** 1H‐NMR spectrum of complex 8 (400 MHz, DMSO‐d6).
**Figure S16:** 13C‐NMR spectrum of complex 8 (100 MHz, DMSO‐d6).

## Data Availability

The data that support the findings of this study are available in the Supporting Information of this article.
