# Supplementary material for: Novel Silver(I) and Gold(I) N‐Heterocyclic Carbene Complexes Induce ROS‐Dependent Autophagic Cell Death in Human Hepatoma Cell Line HepG2
Source: Chem Biol Drug Des. 2026 Mar 27;107(4):e70283. doi: 10.1111/cbdd.70283 (PMC13031429; doi:10.1111/cbdd.70283)
Supplement: Supplementary file 1 — Figure S1: cbdd70283‐sup‐0001‐FigureS1‐S16.doc. 1H‐NMR spectrum of compound A (400 MHz, DMSO‐d6). Figure S2: 13C‐NMR spectrum of compound A (75 MHz, DMSO‐d6). Figure S3: 1H‐NMR spectrum of compound SA (400 MHz, DMSO‐d6). Figure S4: 13C‐NMR spectrum of compound SA (100 MHz, DMSO‐d6). Figure S5: 1H‐NMR spectrum of complex 5 (400 MHz, DMSO‐d6). Figure S6: 13C‐NMR spectrum of complex 5 (100 MHz, DMSO‐d6). Figure S7: 1H‐NMR spectrum of complex 6 (400 MHz, DMSO‐d6). Figure S8: 13C‐NMR spectrum of complex 6 (100 MHz, DMSO‐d6). Figure S9: 1H‐NMR spectrum of compound B (300 MHz, DMSO‐d6). Figure S10: 13C‐NMR spectrum of compound B (75 MHz, DMSO‐d6). Figure S11: 1H‐NMR spectrum of compound SB (300 MHz, DMSO‐d6). Figure S12: 13C‐NMR spectrum of compound SB (100 MHz, DMSO‐d6). Figure S13: 1H‐NMR spectrum of complex 7 (400 MHz, DMSO‐d6). Figure S14: 13C‐NMR spectrum of complex 7 (100 MHz, DMSO‐d6). Figure S15: 1H‐NMR spectrum of complex 8 (400 MHz, DMSO‐d6). Figure S16: 13C‐NMR spectrum of complex 8 (100 MHz, DMSO‐d6). [file CBDD-107-e70283-s001.doc]

**Supporting Information**

**Novel Silver(I) and Gold(I) N-Heterocyclic Carbene Complexes induce ROS-Dependent Autophagic Cell Death in Human Hepatoma cell line HepG2**

**Short running title: Ag/Au-NHCs trigger ROS-based autophagy in HepG2**

**Rocchina Miglionico^1^, Francesco Viceconte^2^, Maria Francesca Armentano^1^,, Annaluisa Mariconda^2^,, Ilaria Nigro^1^, Pasquale Longo^3,#^, Faustino Bisaccia^1, #^**

^1^ Department of Health Sciences, University of Basilicata, Via Dell’Ateneo Lucano 10, 85100 Potenza, Italy.

^2^ Department of Basic and Applied Sciences, University of Basilicata, Via Dell’Ateneo Lucano 10, 85100 Potenza, Italy.

^3^ Department of Chemistry and Biology, University of Salerno, Via Giovanni Paolo II, 132, 84084 Fisciano, Italy

**Correspondence:** Maria Francesca Armentano ([mariafancesca.armentano@unibas.it](mailto:mariafancesca.armentano@unibas.it)) and Annaluisa Mariconda ([annaluisa.mariconda@unibas.it](mailto:annaluisa.mariconda@unibas.it))

^#^ Equal Contribution

**List of Contents**

[**^1^H-NMR of 1-(2-hydroxy-2-phenylethyl)-1H-imidazole (A) 3**](#__RefHeading___Toc172916206)

[**^13^C-NMR of 1-(2-hydroxy-2-phenylethyl)-1H-imidazole (A) 4**](#__RefHeading___Toc172916207)

[**^1^H-NMR of 3-(3-(2-hydroxy-2-phenylethyl)-1H-imidazol-3-ium-1-yl)propane-1-sulfonate (S_A_) 5**](#__RefHeading___Toc172916208)

[**^13^C-NMR 3-(3-(2-hydroxy-2-phenylethyl)-1H-imidazol-3-ium-1-yl)propane-1-sulfonate (S_A_) 6**](#__RefHeading___Toc172916209)

[**^1^H-NMR of disodium bis(1-(2-hydroxy-2-phenylethyl)-3-(3-sulfonatopropyl)-2,3-dihydro-1H-imidazol-2-yl)silver chloride (5) 7**](#__RefHeading___Toc172916210)

[**^13^C-NMR of disodium bis(1-(2-hydroxy-2-phenylethyl)-3-(3-sulfonatopropyl)-2,3-dihydro-1H-imidazol-2-yl)silver chloride (5) 8**](#__RefHeading___Toc172916211)

[**^1^H-NMR of disodium bis(1-(2-hydroxy-2-phenylethyl)-3-(3-sulfonatopropyl)-2,3-dihydro-1H-imidazol-2-yl)gold(I) chloride (6) 9**](#__RefHeading___Toc172916212)

[**^13^C-NMR of disodium bis(1-(2-hydroxy-2-phenylethyl)-3-(3-sulfonatopropyl)-2,3-dihydro-1H-imidazol-2-yl)gold(I) chloride (6) 10**](#__RefHeading___Toc172916213)

[**^1^H-NMR of 1-(2-hydroxy-2-phenylethyl)-1H-4,5-dichloroimidazole (B) 11**](#__RefHeading___Toc172916214)

[**^13^C-NMR 1-(2-hydroxy-2-phenylethyl)-1H-4,5-dichloroimidazole (B) 12**](#__RefHeading___Toc172916215)

[**^1^H-NMR of 3-(4,5-dichloro-3-(2-hydroxy-2-phenylethyl)-1H-imidazol-3-ium-1-yl)propane-1-sulfonate (S_B_) 13**](#__RefHeading___Toc172916216)

[**^13^C-NMR of 3-(4,5-dichloro-3-(2-hydroxy-2-phenylethyl)-1H-imidazol-3-ium-1-yl)propane-1-sulfonate (S_B_) 14**](#__RefHeading___Toc172916217)

[**^1^H-NMR of sodium bis(4,5-dichloro-1-(2-hydroxy-2-phenylethyl)-3-(3-sulfonatopropyl)-2,3-dihydro-^1^H-imidazol-2-yl)silver(I) chloride (7) 15**](#__RefHeading___Toc172916218)

[**^13^C-NMR of sodium bis(4,5-dichloro-1-(2-hydroxy-2-phenylethyl)-3-(3-sulfonatopropyl)-2,3-dihydro-^1^H-imidazol-2-yl)silver(I) chloride (7) 16**](#__RefHeading___Toc172916219)

[**^1^H-NMR of sodium bis(4,5-dichloro-1-(2-hydroxy-2-phenylethyl)-3-(3-sulfonatopropyl)-2,3-dihydro-1H-imidazol-2-yl)gold(I) chloride (8) 17**](#__RefHeading___Toc172916220)

[**^13^C-NMR of sodium bis(4,5-dichloro-1-(2-hydroxy-2-phenylethyl)-3-(3-sulfonatopropyl)-2,3-dihydro-^1^H-imidazol-2-yl)gold(I) chloride (8) 18**](#__RefHeading___Toc172916221)

**^1^H-NMR of 1-(2-hydroxy-2-phenylethyl)-1H-imidazole (A)**


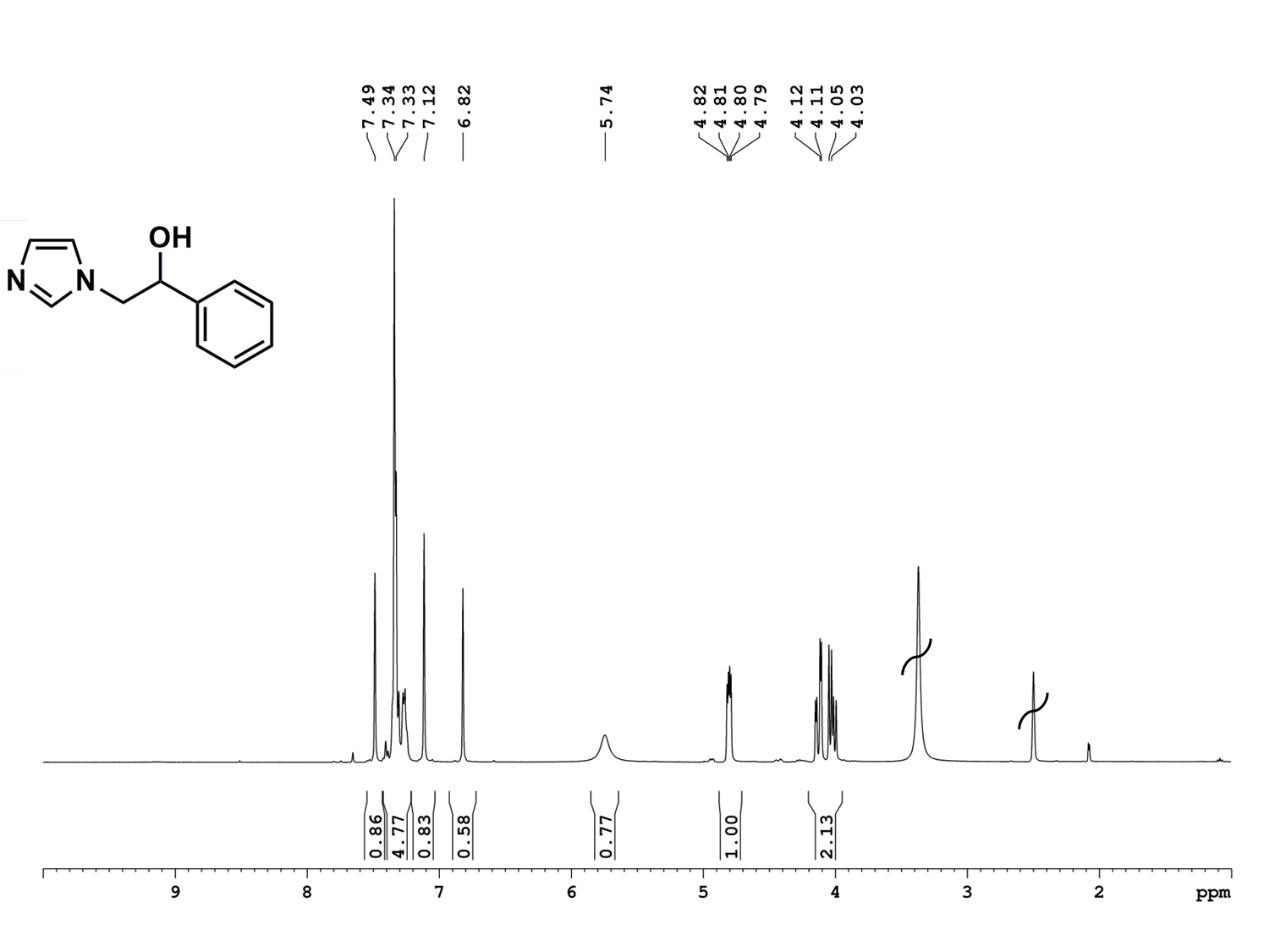

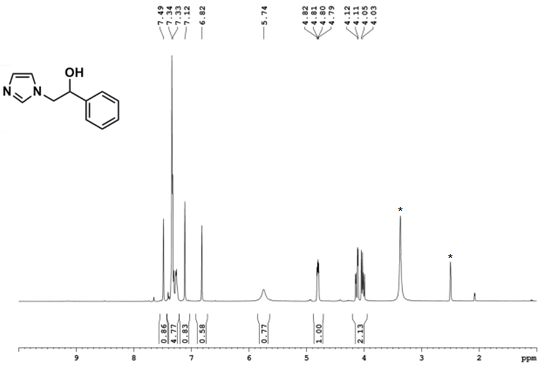


**Fig. S1**: ^1^H-NMR spectrum of A

*due to solvents

**^1^H-NMR** (400 MHz, DMSO-*d_6_*, δ ppm): 7.45 (s, 1H, NC***H***N,), 7.28 (m, 5H, aromatic protons), 7.05 (s, 1H, CH_2_NC***H***CHN,), 6.78 (s, CH_2_NCHC***H***N, 1H), 5.74 (br, 1H, O***H***), 4.78 (m, 1H, C***H***OH,), 4.18-3.98 (m, 2H, NC***H_2_***CHOH,).

# ^13^C-NMR of 1-(2-hydroxy-2-phenylethyl)-1H-imidazole (A)


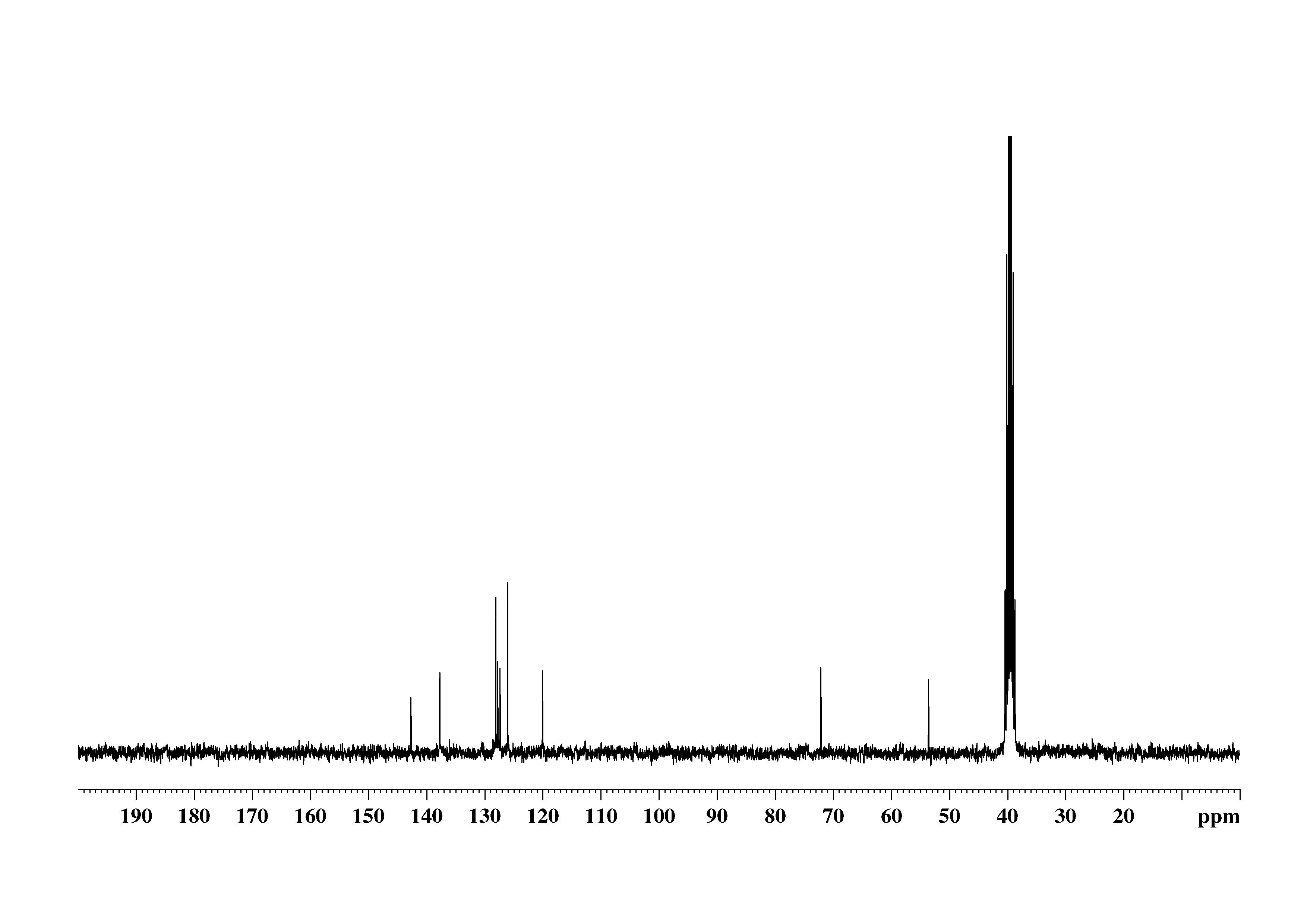


**Fig. S2**: ^13^C-NMR spectrum of A

**^13^C-NMR** (75 MHz, DMSO-*d_6_*, δ ppm): 142.6 (ipso aromatic carbon), 137.7 (N***C***HN), 128.0-127.7-127.3-126.0 (aromatic carbons), 120.0 (N***C***H***C***HN), 72.0 (***C***HOH), 53.5 (N***C***H_2_).

# ^1^H-NMR of 3-(3-(2-hydroxy-2-phenylethyl)-1H-imidazol-3-ium-1-yl)propane-1-sulfonate (S_A_)


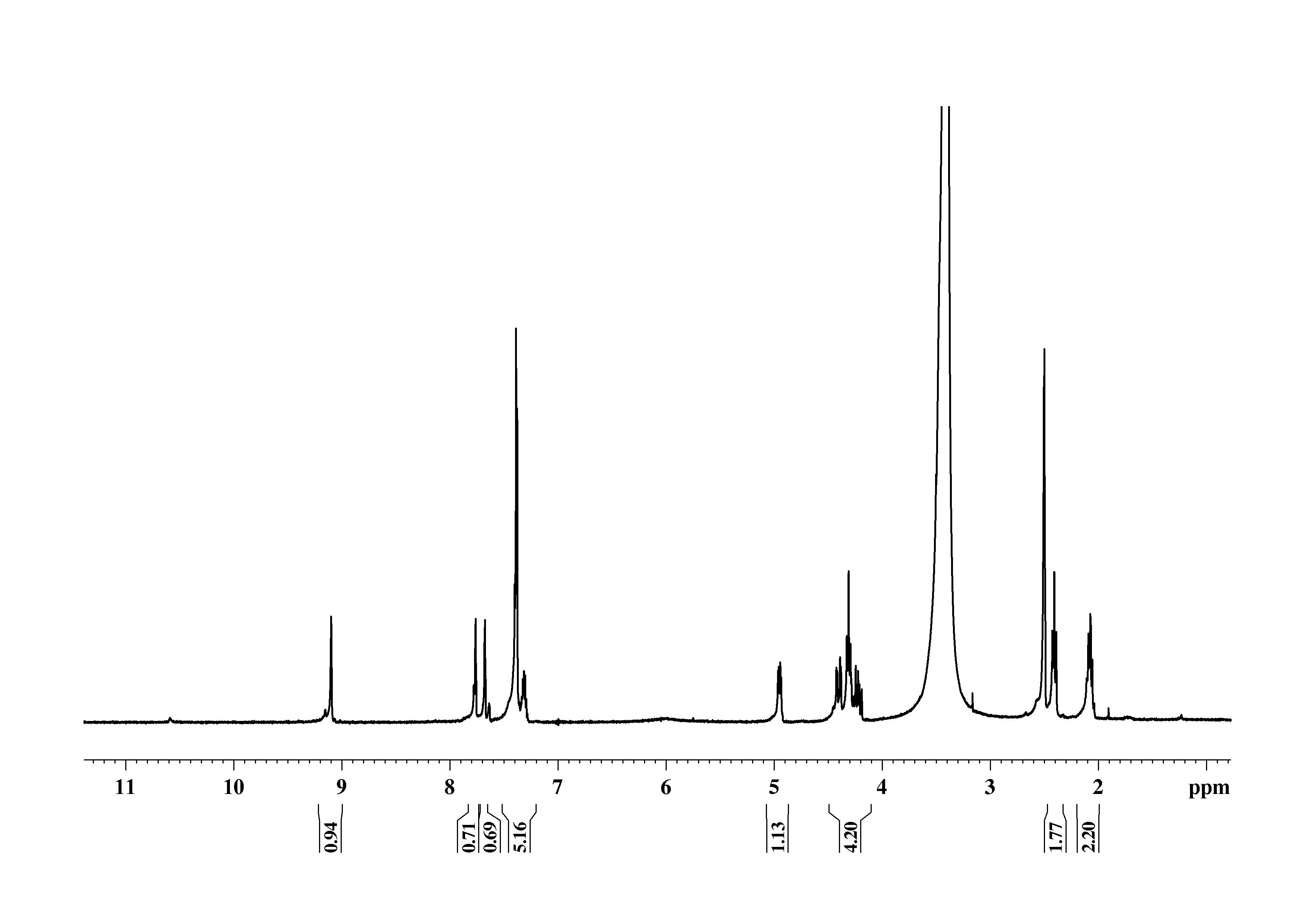


**Fig. S3**: ^1^H-NMR spectrum of S_A_

**^1^H-NMR** (400 MHz, DMSO-*d_6_*, δ ppm): 9.10 (s, 1H, NC***H***N), 7.77 (s, 1H, CHCH_2_NC***H***CHNCH_2_CH_2_,), 7.68 (s, 1H, CHCH_2_NCHC***H***NCH_2_CH_2_), 7.39 (m, 5H, aromatic protons), 4.94 (m, 1H, C***H***OH)), 4.42-4.19 (m, 4H, CHC***H_2_***NCH and NC***H_2_***CH_2_CH_2_), 2.11 (t, 2H, NCH_2_CH_2_C***H_2_***), 2.07 (m, 2H, NCH_2_C***H_2_***CH_2_).

# ^13^C-NMR 3-(3-(2-hydroxy-2-phenylethyl)-1H-imidazol-3-ium-1-yl)propane-1-sulfonate (S_A_)


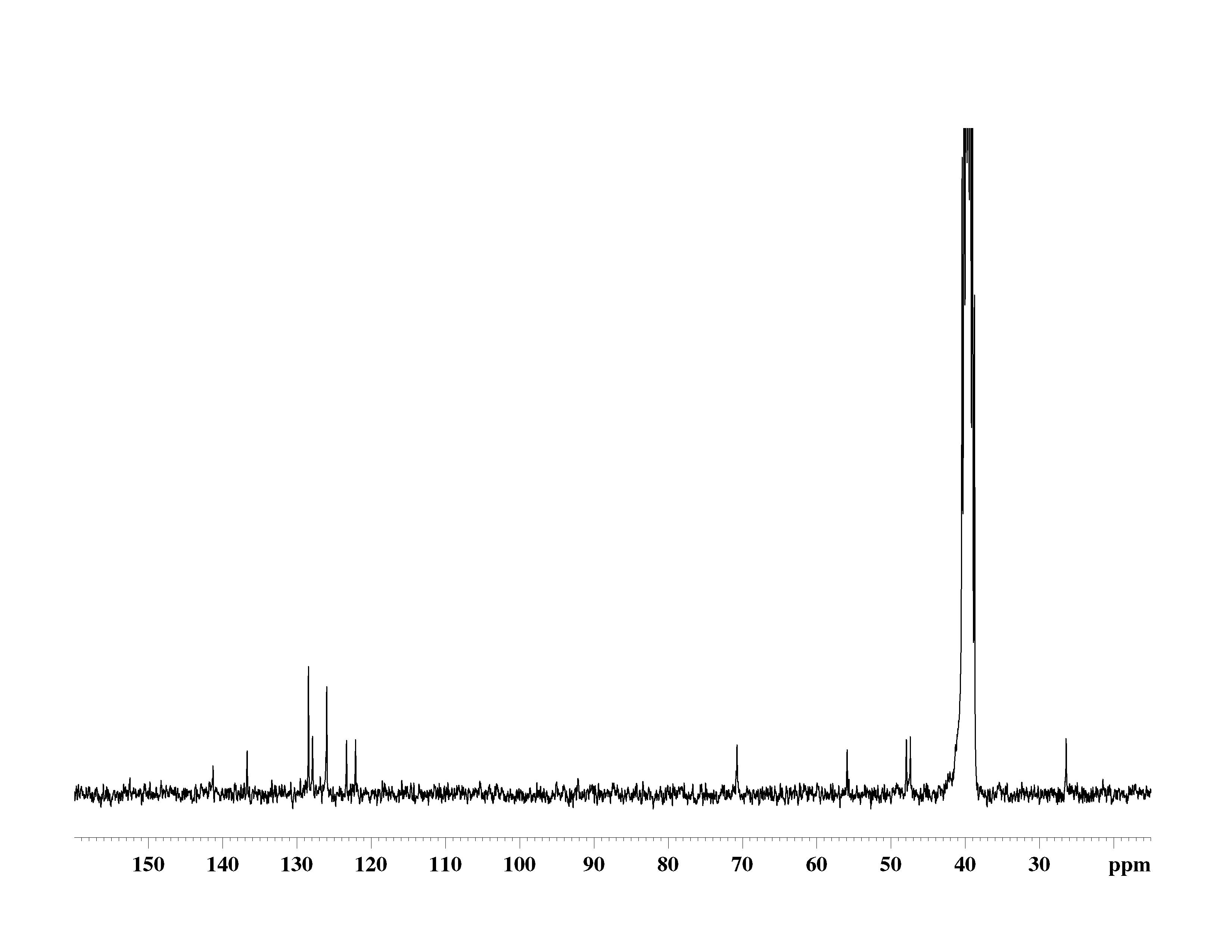


**Fig. S4**: ^13^C-NMR spectrum of S_A_

**^13^C-NMR** (100 MHz, DMSO-*d_6_*, δ ppm): 141.6 (ipso aromatic carbon), 137.0 (N***C***HN), 128.7, 128.2, 126.3 (aromatic carbons), 123.6 and 122.4 (N***C***H***C***HN), 71.0 (***C***HOH), 56.2 (N***C***H_2_CHOH), 48.2 (N***C***H_2_CH_2_CH_2_), 47.7 (NCH_2_CH_2_***C***H_2_), 26.7 (NCH_2_***C***H_2_CH_2_).

Elemental Analysis: calculated for C_14_H_18_N_2_O_4_S C, 54.18; H, 5.85; N, 9.03. Found C, 54.19; H, 5.85; N, 9.02.

(MALDI-TOF, CH_3_OH): m/z = 309.16020 Dalton attributable to [C_14_H_17_N_2_O_4_S]^+^.

**^1^H-NMR of disodium bis(1-(2-hydroxy-2-phenylethyl)-3-(3-sulfonatopropyl)-2,3-dihydro-1H-imidazol-2-yl)silver chloride (5)**


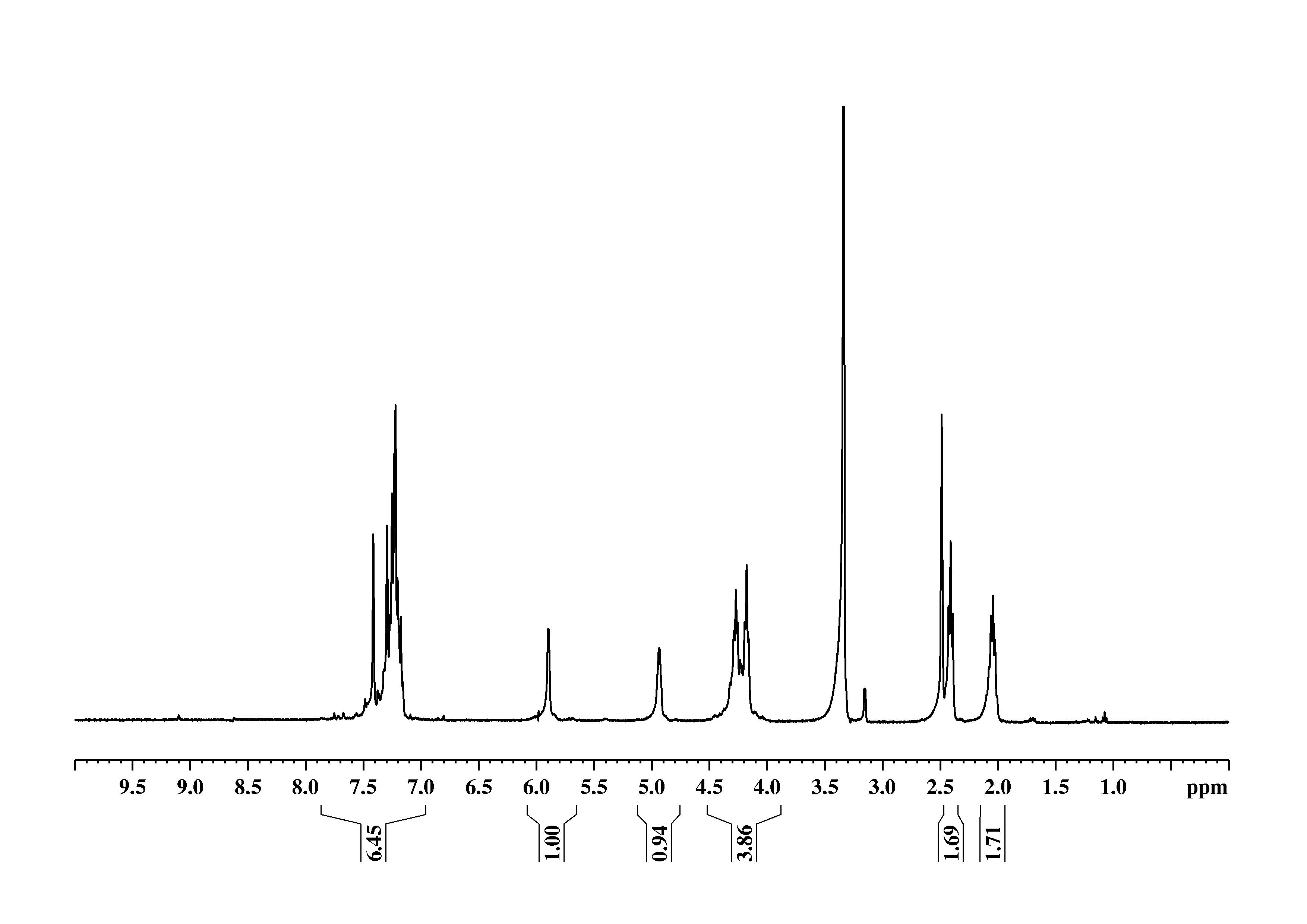


**Fig. S5**: ^1^H-NMR spectrum of 5

**^1^H-NMR** (400 MHz, DMSO-*d_6_*, δ ppm): 7.49-7.21 (m, 7H, aromatic protons + NC***H***C***H***N), 5.93 (s, 1H, O***H***), 4.94 (br, 1H, C***H***OH), 4.27-4.18 (m, 4H, NC***H_2_***CH and NC***H_2_***CH_2_CH_2_), 2.40 (t, 2H, NCH_2_CH_2_C***H_2_***), 2.06 (m, 2H, NCH_2_C***H_2_***CH_2_).

**^13^C-NMR of disodium bis(1-(2-hydroxy-2-phenylethyl)-3-(3-sulfonatopropyl)-2,3-dihydro-1H-imidazol-2-yl)silver chloride (5)**


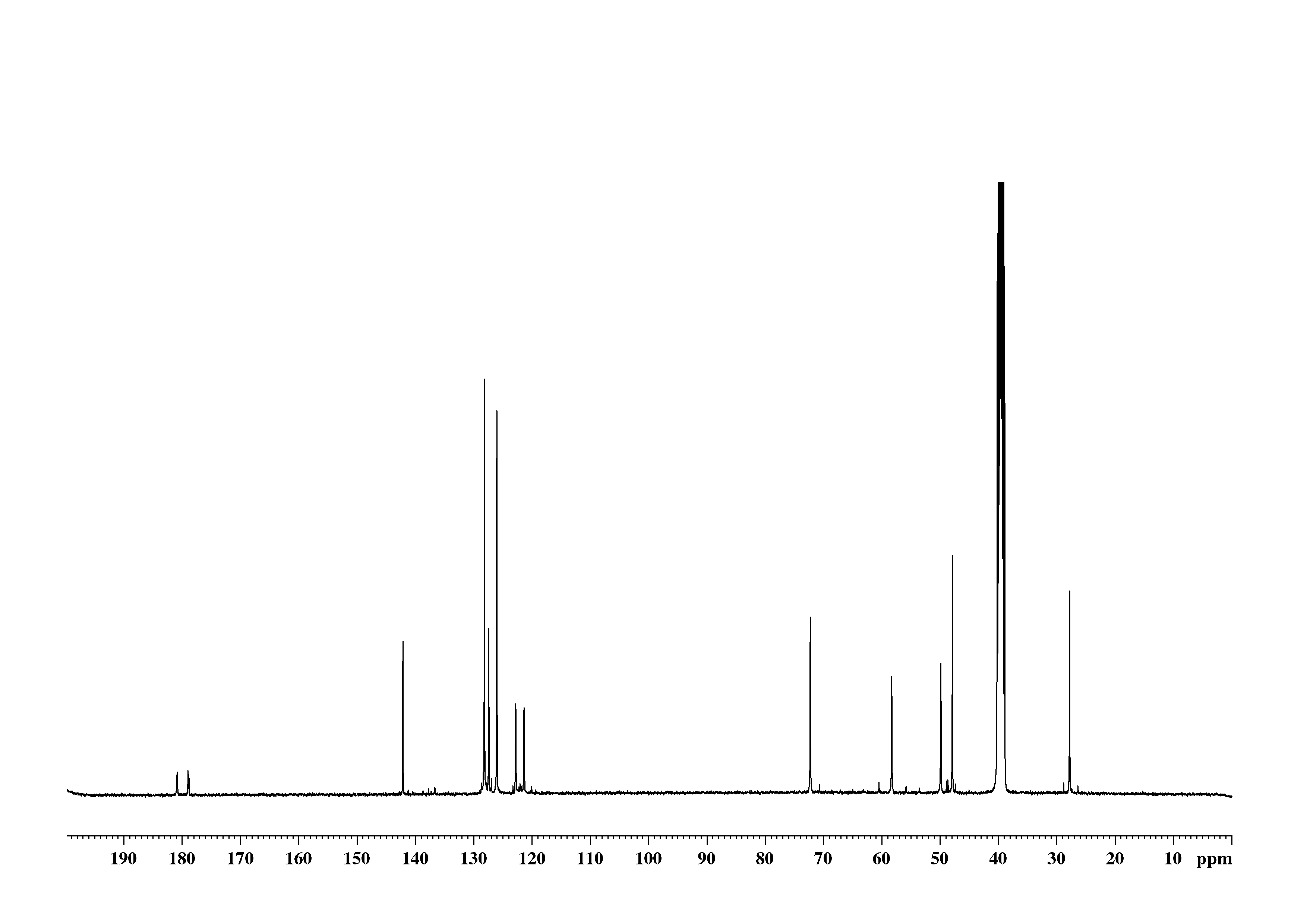


**Fig. S6**: ^13^C-NMR spectrum of 5

**^13^C-NMR** (100 MHz, DMSO-*d_6_*, δ ppm): 180.8 and 178.9 (dd, 107/109Ag, N***C***N), 142.1 (ipso aromatic carbon), 128.1, 127.4, 126.0 (aromatic carbons), 122.8 and 121.3 (N***C***H***C***HN), 72.2 (***C***HOH), 58.2 (N***C***H_2_CHOH), 49.8 (N***C***H_2_CH_2_CH_2_), 47.8 (NCH_2_CH_2_***C***H_2_), 27.2 (NCH_2_***C***H_2_CH_2_).

Elemental Analysis: calculated for C_28_H_34_AgClN_4_Na_2_O_8_S_2_: C, 41.62; H, 4.24; N, 6.93. Found C, 41.62; H, 4.25; N, 6.92.

(MALDI-TOF, DHBA): m/z = 771.06754 Dalton attributable to [C_28_H_34_AgN_4_Na_2_O_8_S_2_]^+^; 749.08562 Da attributable to [C_28_H_34_AgN_4_NaO_8_S_2_]^+^; 727.10342 Da attributable to [C_28_H_35_AgN_4_O_8_S_2_]^+^.

**^1^H-NMR of** **disodium bis(1-(2-hydroxy-2-phenylethyl)-3-(3-sulfonatopropyl)-2,3-dihydro-1H-imidazol-2-yl)gold(I) chloride (6)**


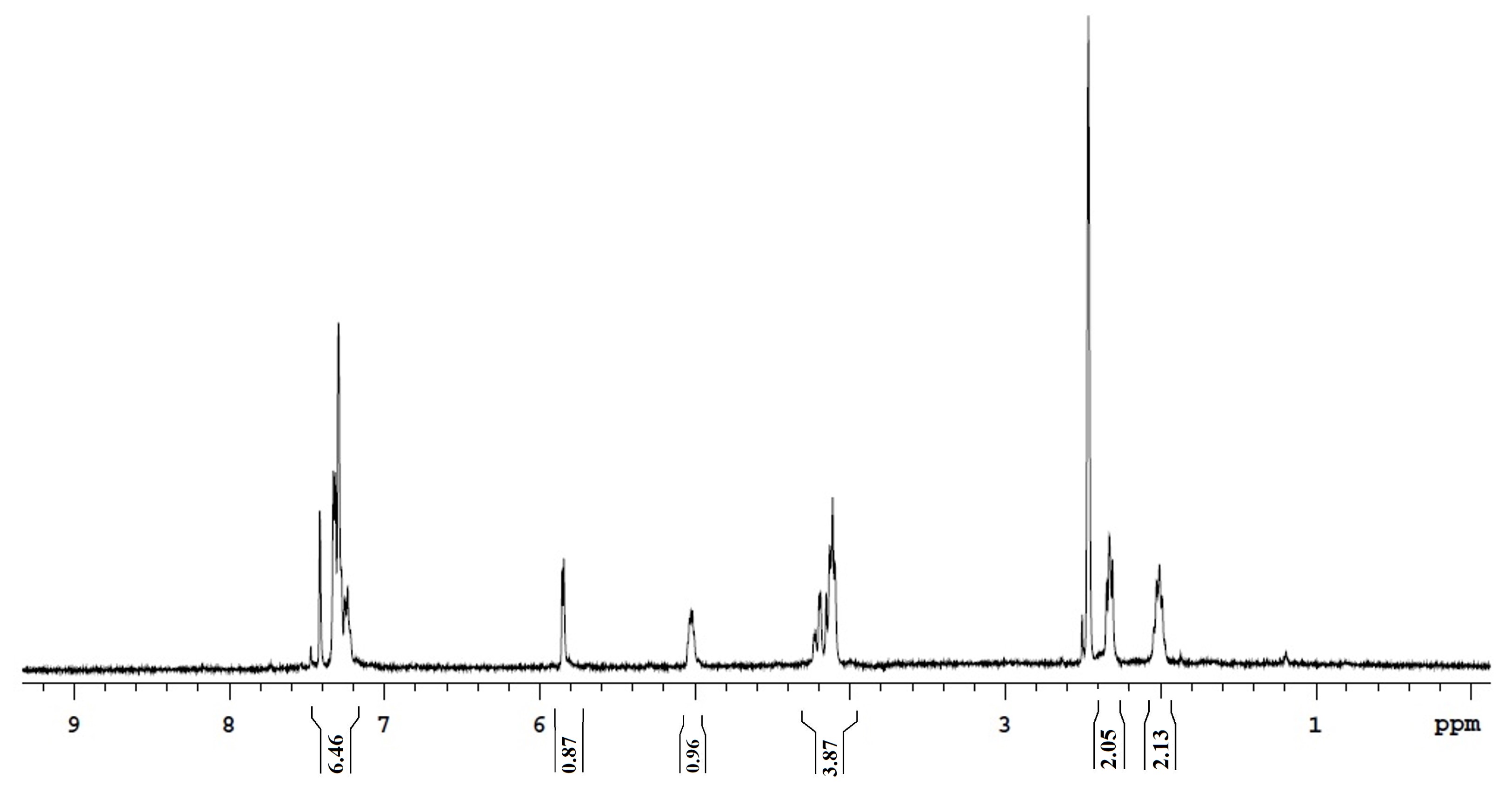


**Fig. S7**: ^1^H-NMR spectrum of 6

**^1^H-NMR** (400 MHz, DMSO-*d_6_*, δ ppm): 7.41-7.23 (m, 7H, aromatic protons + NC***H***C***H***N), 5.84 (d, 1H, O***H***), 5.04 (m, C***H***OH, 1H), 4.23-4.14 (m, 4H, NC***H_2_***CH and NC***H_2_***CH_2_CH_2_), 2.32 (t, 2H, NCH_2_CH_2_C***H_2_***), 2.02 (m, H, NCH_2_C***H_2_***CH_2_).

**^13^C-NMR of disodium bis(1-(2-hydroxy-2-phenylethyl)-3-(3-sulfonatopropyl)-2,3-dihydro-1H-imidazol-2-yl)gold(I) chloride (6)**


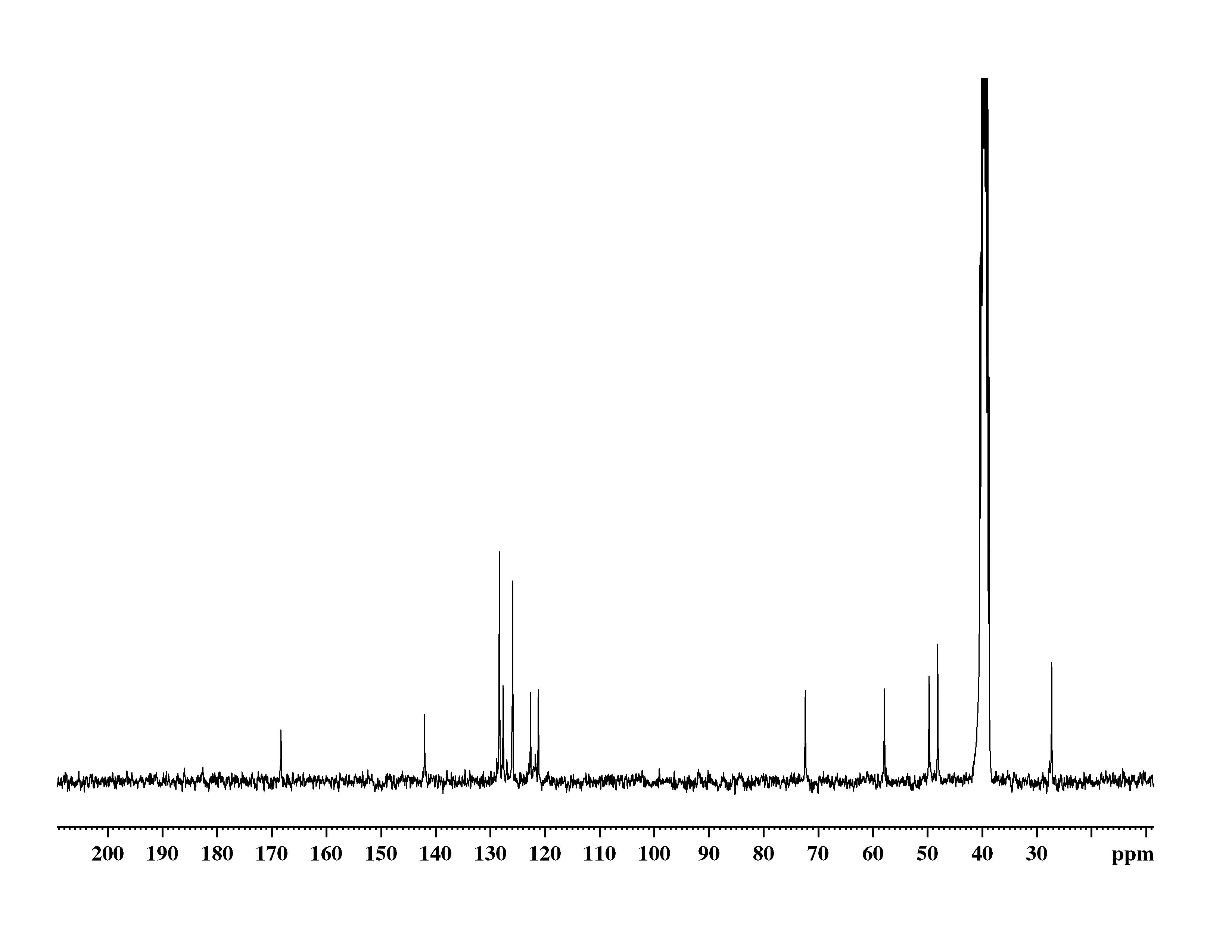


**Fig. S8**: ^13^C-NMR spectrum of 6

**^13^C-NMR** (100 MHz, DMSO-*d_6_*, δ ppm): 168.3 (N***C***N), 142.3 (ipso aromatic carbon), 128.2, 126.9, 125.5 (aromatic carbons), 123.3 and 121.2 (N***C***H***C***HN), 72.2 (***C***HOH), 58.4 (N***C***H_2_CHOH), 48.8 (N***C***H_2_CH_2_CH_2_), 48.2 (NCH_2_CH_2_***C***H_2_), 27.1 (NCH_2_***C***H_2_CH_2_).

Elemental Analysis: calculated for C_28_H_34_AuClN_4_Na_2_O_8_S_2_: C, 37.49; H, 3.82; N, 6.25. Found C, 37.50; H, 3.82; N, 6.24.

(MALDI-TOF, DIBAH): m/z = 861.13148 Da attributable to [C_28_H_34_AuN_4_Na_2_O_8_S_2_]^+^; 506.53205 Da attributable to [C_14_H_17_AuN_2_O_4_S]^+^.

**^1^H-NMR of** **1-(2-hydroxy-2-phenylethyl)-1H-4,5-dichloroimidazole (B)**

**
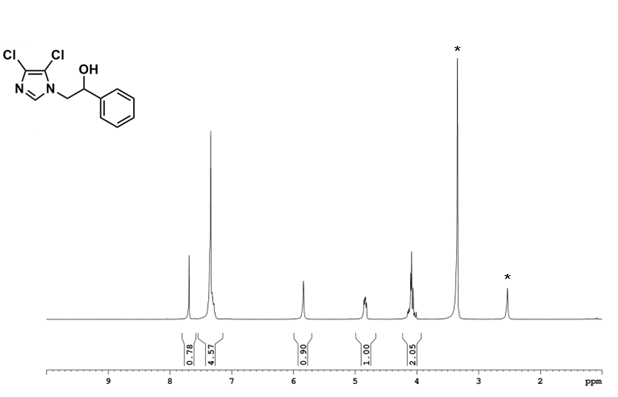
**

***Fig. S9****: ^1^H-NMR spectrum of B*

*due to solvents

**^1^H-NMR** (300 MHz, DMSO-*d_6_*, δ ppm): 7.69 (s, 1H, NC***H***N) 7.34-7.31 (m, 5H, aromatic protons), 5.83 (d, 1H, O***H***), 4.86 (m, 1H, C***H***OH), 4.10 (m, 2H, NC***H_2_***CHOH).

**^13^C-NMR 1-(2-hydroxy-2-phenylethyl)-1H-4,5-dichloroimidazole (B)**

**Fig. S10**: ^13^C-NMR spectrum of B

**^13^C-NMR** (75 MHz, DMSO-*d6*, δ ppm): 141.72 (ipso aromatic carbon), 136.71 (N***C***HN), 128.26, 127.68, 125.92, (aromatic carbons), 123.77 and 112.42 (N***C***Cl***C***ClN), 70.77 (***C***HOH), 52.71 (N***C***H2CHOH).

**^1^H-NMR of 3-(4,5-dichloro-3-(2-hydroxy-2-phenylethyl)-1H-imidazol-3-ium-1-yl)propane-1-sulfonate (S_B_)**


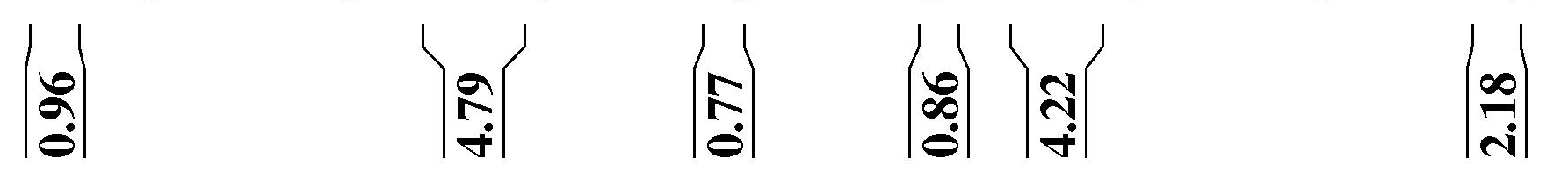


**Fig. S11**: ^1^H-NMR spectrum of S_B_

**^1^H-NMR** (300 MHz, DMSO-*d6*, δ ppm): 9.51 (s, 1H, NC***H***N), 7.39 (m, 5H, aromatic protons), 6.08 (d, 1H, O***H***), 4.94 (m, 1H, C***H***OH), 4.43-4.27 (m, 4H, NC***H2***CHOH and NC***H2***CH2), 2.52 (m, 2H, NCH2CH2C***H2***), 2.09 (m, 2H, NCH2C***H2***CH2).

**^13^C-NMR of 3-(4,5-dichloro-3-(2-hydroxy-2-phenylethyl)-1H-imidazol-3-ium-1-yl)propane-1-sulfonate (S_B_)**

**Fig. S12**: ^13^C-NMR spectrum of S_B_

**^13^C-NMR** (100 MHz, DMSO-d_6_, δ ppm): 140.38 (ipso aromatic carbon), 137.10 (N**C**HN), 128.5, 128.09, 125.92 (aromatic carbons), 119.12 and 118.24 (N**C**Cl**C**ClN), 69.81 (**C**HOH), 55.05 (N**C**H_2_CHOH), 47.69 (NCH_2_CH_2_**C**H_2_), 47.18 (NCH_2_CH_2_**C**H_2_), 24.88 (NCH_2_**C**H_2_CH_2_).

Elemental Analysis: calculated for C_14_H_16_Cl_2_N_2_O_4_S C, 44.34; H, 4.25; N, 7.39. Found C, 44.39; H, 4.18; N, 7.45.

(MALDI-TOF, CH_3_OH): m/z 378.02073 Dalton attributable to [C_12_H_16_Cl_2_N_2_O_4_S]^+^.

**^1^H-NMR of** **sodium bis(4,5-dichloro-1-(2-hydroxy-2-phenylethyl)-3-(3-sulfonatopropyl)-2,3-dihydro-1H-imidazol-2-yl)silver(I) chloride (7)**


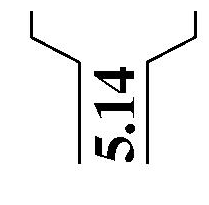

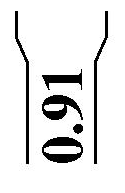

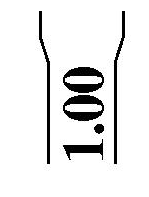

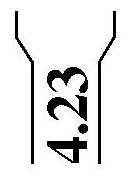

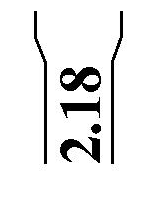

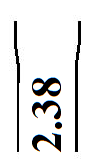

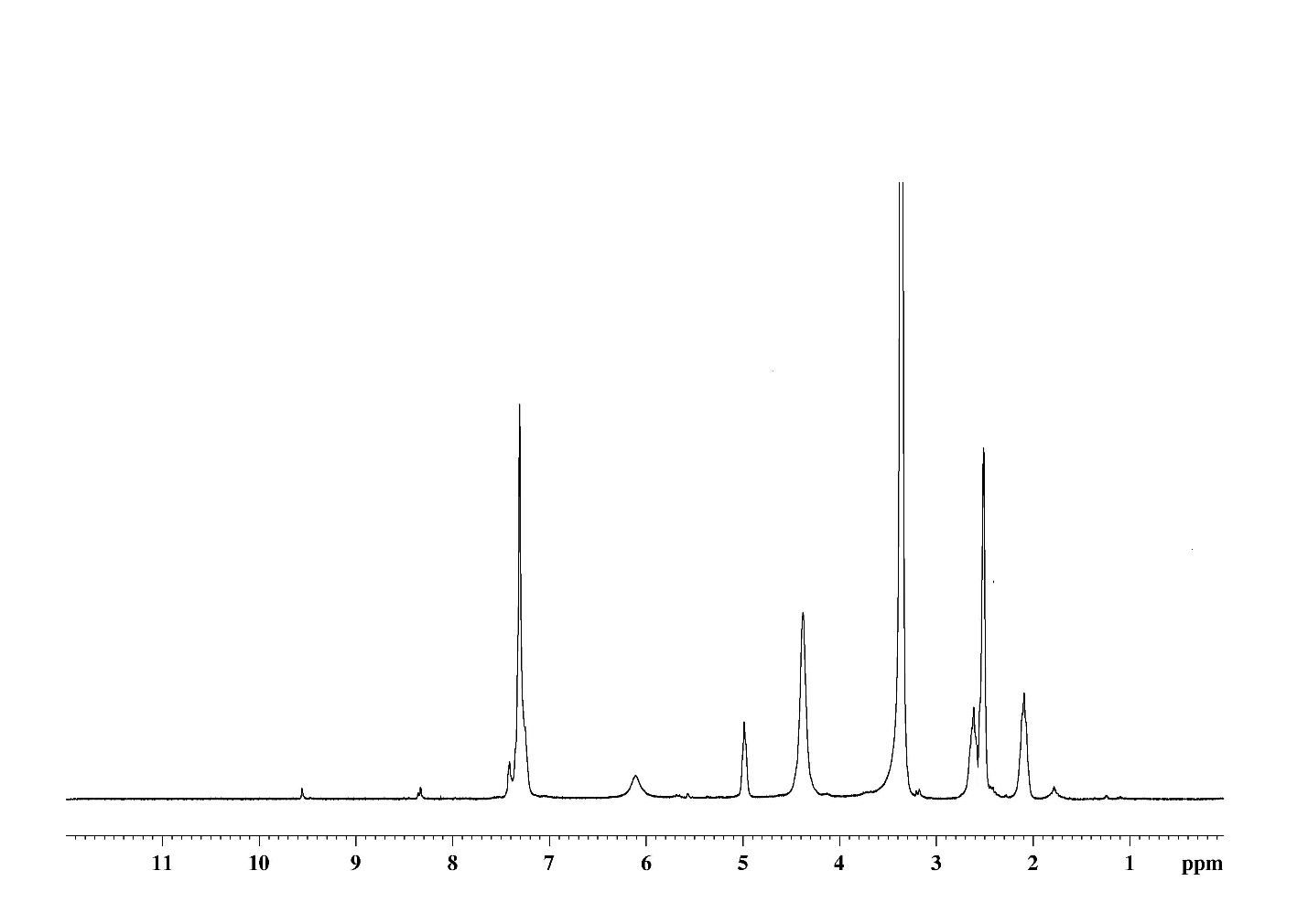


**Fig. S13**: ^1^H-NMR spectrum of 7

**^1^H-NMR** (400 MHz, DMSO-*d_6_*, δ ppm): 7.30 (m, 5H, aromatic protons), 6.10 (b, 1H, O***H***), 4.98 (br, 1H, C***H***OH), 4.37 (br, 4H, NC***H_2_***CHOH e NC***H_2_***CH_2_CH_2_), 2.57 (m, 2H, NCH_2_CH_2_C***H_2_***), 2.09 (br, 2H, NCH_2_C***H_2_***CH_2_).

**^13^C-NMR of sodium bis(4,5-dichloro-1-(2-hydroxy-2-phenylethyl)-3-(3-sulfonatopropyl)-2,3-dihydro-1H-imidazol-2-yl)silver(I) chloride (7)**


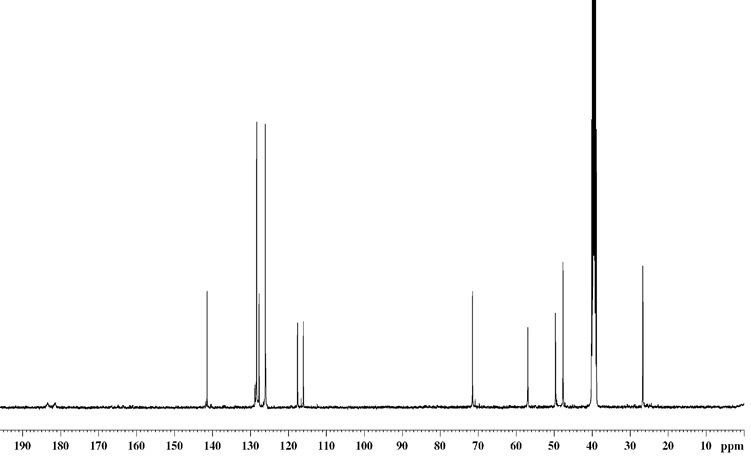


**Fig. S14**: ^13^C-NMR spectrum of 7

**^13^C-NMR** (100 MHz, DMSO-*d_6_*, δ ppm): 183.66 and 181.68 (dd, 107/109Ag, NCN), 141.43 (ipso aromatic carbon), 128.36, 127.73, 126.09 (aromatic carbons), 117.58 and 116.07 (N***C***Cl***C***ClN), 71.51 (***C***HOH), 56.69 (N***C***H_2_CHOH), 48.62 (N***C***H_2_CH_2_CH_2_), 47.71 (NCH_2_CH_2_**C**H_2_), 26.67 (NCH_2_CH_2_***C***H_2_).

Elemental Analysis: calculated for C_30_H_38_AuClN_4_Na_2_O_8_S_2_ C, 38.95; H, 4.14; N, 6.06. Found C, 38.99; H, 4.20; N, 6.01.

MALDI-TOF (CH3OH, m/z) = 781.05139 Dalton attributable to [C28H30Cl4N4O5AgS]^+^ e 401.01253 Dalton attributable to [C14H16Cl2N2O4SNa]^+^.

**^1^H-NMR of sodium bis(4,5-dichloro-1-(2-hydroxy-2-phenylethyl)-3-(3-sulfonatopropyl)-2,3-dihydro-1H-imidazol-2-yl)gold(I) chloride (8)**


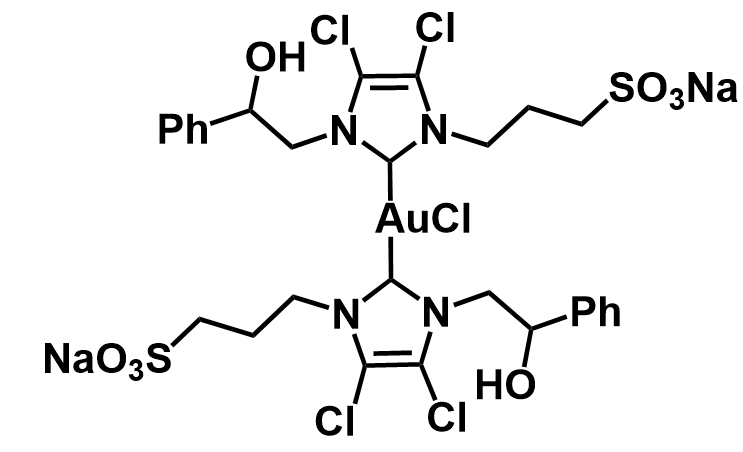

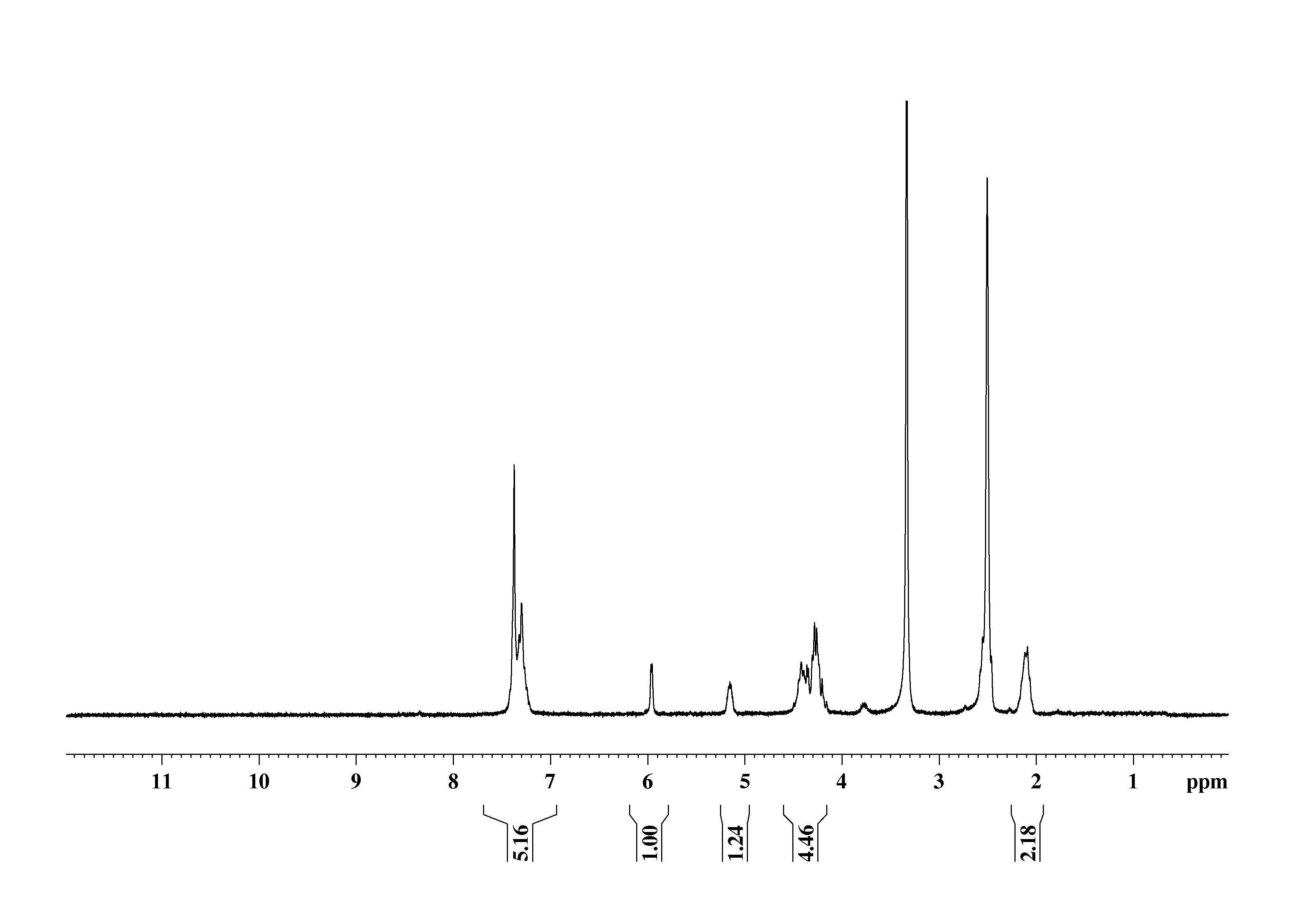


**Fig. S15**: AuBG2 ^1^H-NMR spectrum of 8

**^1^H-NMR** (400 MHz, DMSO-*d_6_*, δ ppm): 7.37-7.29 (m, 5H, aromatic protons), 6.09 (s, 1H, O***H***) 5.15 (m, 1H, C***H***OH), 4.30 (m, 4H, NC***H_2_***CHOH e NC***H_2_***CH_2_CH_2_), 2.55 (o, 2H, NCH_2_CH_2_C***H_2_***), 2.11 (m, 2H, NCH_2_C***H_2_***CH_2_).

**^13^C-NMR of sodium** **bis(4,5-dichloro-1-(2-hydroxy-2-phenylethyl)-3-(3-sulfonatopropyl)-2,3-dihydro-1H-imidazol-2-yl)gold(I) chloride (8)**


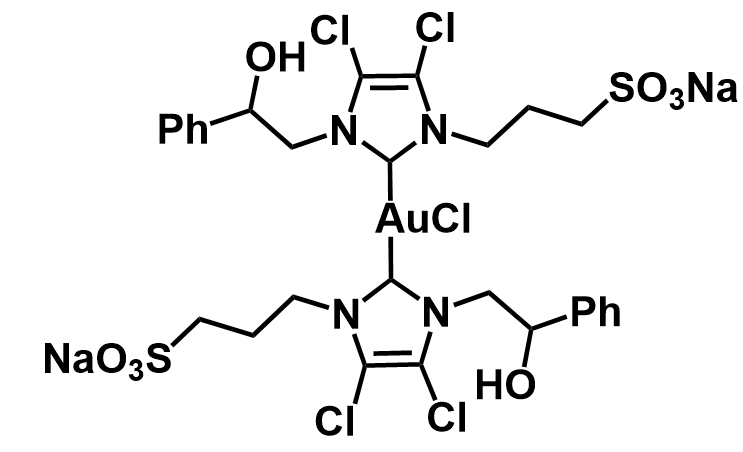


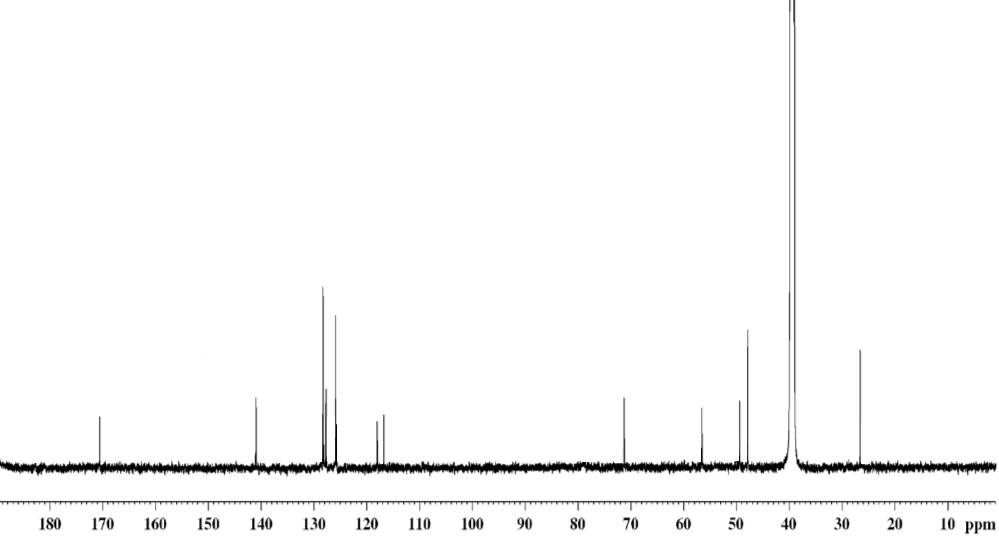


**Fig. S16**: AuBG2 ^13^C-NMR spectrum of 8

**^13^C-NMR** (100 MHz, DMSO-*d_6_*, δ ppm): 170.08 (N**C**N), 141.00 (ipso aromatic carbon), 128.39, 128.18, 125.97 (aromatic carbons), 118.06 and 116.81 (N***C***Cl***C***ClN), 71.12 (***C***HOH), 56.79 (N***C***H_2_CHOH), 49.42 (N**C**H_2_CH_2_CH_2_), 47.89 (NCH_2_CH_2_***C***H_2_), 26.62 (NCH_2_***C***H_2_CH_2_).

Elemental Analysis: calculated for C_28_H_30_AuCl_5_N_4_Na_2_O_8_S_2_ C, 32.50; H, 2.92; N, 5.41. Found C, 32.59; H, 3.01; N, 5.49.

MALDI-TOF (CH_3_OH, m/z): 501.03867 Dalton, attributable to [C_8_H_10_Cl_2_N_2_O_4_AuS]^+^ and 483.02756 Dalton attributable to [C_8_H_9_Cl_2_N_2_O_3_AuS]^+^.
